# Surgery for pineal cysts with symptoms in the absence of hydrocephalus: a prospective cohort study

**DOI:** 10.1016/j.eclinm.2025.103514

**Published:** 2025-09-19

**Authors:** Riccardo Masina, Jessica Harding, Wendi Qian, Tomasz Matys, Maria Harrington, Anna Hill, Jeff Faux, Christopher Quelch, Ihor Tysh, Thanasis Paschalis, Amber Steele, Kieren Allinson, Alexis Joannides, Angelos Kolias, Martin Taphoorn, Linda Dirven, Thomas Santarius

**Affiliations:** a Department of Neurosurgery, Addenbrooke's Hospital, Cambridge University Hospitals, Cambridge, UK; b Department of Clinical Neurosciences, University of Cambridge, Cambridge, UK; c Department of Physiology, Development, and Neuroscience, University of Cambridge, Cambridge, UK; d Department of Radiology, University of Cambridge, Cambridge, UK; e Pineal Cyst, UK; f Research Strategy & Partnership Hub, Cambridge NIHR Biomedical Research Centre, Cambridge, UK; g Department of Neurology, Leiden University Medical Center, Leiden, Netherlands; h Department of Pathology, Addenbrooke's Hospital, Cambridge University Hospitals, Cambridge, UK; i Cambridge Cancer Trials Centre, Cambridge Clinical Trials Unit – Cancer Theme, Cambridge University Hospitals NHS Foundation Trust, Cambridge, UK

**Keywords:** Pineal cyst, Neurosurgery, Prospective cohort study, Health-related quality of life

## Abstract

**Background:**

There is mounting evidence for surgery as an effective treatment in selected patients with non-hydrocephalic symptomatic pineal cyst (nhSPC) syndrome. We present the first prospective cohort study of surgical PC resection to treat nhSPC syndrome.

**Methods:**

CamProS-PC is an observational, single-centre, prospective cohort study. Patients were eligible if aged >18 years, PC size >10 mm, had severe symptoms refractory to medical treatment, without ventriculomegaly. Patient-reported data were collected preoperatively, and 3 and 12 months postoperatively. MR imaging was performed before and 12 months after surgery. The primary outcome was improvement in Role Functioning (RF) at 12 months. Secondary outcomes were changes in other domains of Health-Related Quality of Life (HRQoL) and symptoms at 3 and 12 months, and safety of the intervention. CamProS-PC is registered (ISRCTN51545574) and has been completed.

**Findings:**

Between January 2019 and May 2023, 122 consecutive patients were screened and 40 were recruited and underwent PC resection. No loss of follow-up occurred. Mean age was 38 [SD 28–49] with 80% (32/40) females. At baseline, all patients reported headaches, 95% (38/40) dizziness, and 98% (39/40) reported impairment of vision, 98% (38/40) sleep, 90% (36/40) concentration, 88% (35/40) memory, 68% (27/40) speech, and 60% (24/40) hearing. At 12 months postoperatively, HRQoL was improved across all functional scales: RF mean difference 46 [SD 11–80, p < 0.0001] points, Physical 22 [SD −1 to 45, p < 0.0001], Emotional 35 [SD −2 to 71, p < 0.0001], Cognitive 38 [SD 3–73, p < 0.0001], and Social 50 [SD 14–87, p < 0.0001]. Global Health Status improved by 32 [SD 4–61, p < 0.0001] points. Symptoms improved overall in 95% (38/40) of patients. Most benefits were already seen at 3 months. Complications occurred in 23% (9/40) of patients; one was permanent (diplopia). All patients were alive at last follow-up.

**Interpretation:**

CamProS-PC demonstrated significant benefit to HRQoL and symptoms one year after PC resection with overall acceptable safety profile.

**Funding:**

Department of Clinical Neuroscience, 10.13039/501100000735University of Cambridge.

**Research in context**

**Evidence before this study**

A growing body of evidence describes a subset of pineal cyst (PC) patients that are symptomatic despite the absence of obstructive hydrocephalus. Recently, a number of neurosurgical centres have begun offering cyst resection as a treatment option for these selected patients with non-hydrocephalic symptomatic pineal cyst (nhSPC). The absence of high-quality evidence to assess the efficacy and safety of surgical intervention creates uncertainty for patients and healthcare providers.

In March 2021 we systematically searched PubMed and SCOPUS for all studies reporting surgical resection of pineal cysts in symptomatic patients without ventriculomegaly, using the search strategy detailed in the Appendix (pp. 68–70). Of the 1537 hits identified, 26 reports met inclusion and exclusion criteria. Our published meta-analysis of this cohort of 297 patients supported the safety and efficacy of surgical intervention, but it consisted entirely of case reports and retrospective case series, which are subject to significant bias (PMID: 34854993).

**Added value of this study**

We present the first prospective cohort study of safety and efficacy of surgical management of patients with nhSPC. This study also demonstrates the feasibility of conducting a definitive randomised controlled trial (RCT).

**Implications of all the available evidence**

This prospective cohort study of surgical resection to treat nhSPC met its primary objective. The findings demonstrate significant symptomatic improvement with morbidity and mortality rates comparable to those of previously reported retrospective studies. These results justify a randomised, placebo-controlled trial to compare the safety and efficacy of surgical resection with conservative management.

## Introduction

Pineal cysts (PCs) are the most common midline brain lesion.^1^^,^^2^ They are located in the quadrigeminal cistern, with the anterior wall variably protruding into the third ventricle.^1^^,^^3^ They are histologically benign.^3^ Female-to-male ratio is 1.7, and the prevalence of PCs in autopsy studies ranges between 25% and 40%.^1^^,^^3^ The steady increase in PC detection since the advent of MRI imaging poses a considerable challenge to general and specialist medical practitioners, especially neurologists and neurosurgeons.^2^^,^^4^

Rarely, PCs present with Parinaud's syndrome and obstructive hydrocephalus requiring urgent neurosurgical intervention.^2^ Historically, it has been assumed that all other PCs are asymptomatic. However, a growing body of medical literature describes a subset of PC patients that are symptomatic despite the absence of ventriculomegaly.^5^^,^^6^ This non-hydrocephalic symptomatic PC (nhSPC) syndrome is characterised by headaches, nausea and vomiting, visual disturbances, gait instability, fatigue and hypersomnolence.^5^^,^^7^ The pathophysiology of the syndrome is yet largely unknown. Several mechanisms have been suggested, including crowding of the pineal recess, impairment of the deep venous system outflow, tectal compression, and intermittent cerebrospinal fluid (CSF) obstruction at the aqueduct.^5^, ^6^, ^7^, ^8^, ^9^, ^10^, ^11^, ^12^

The relatively nonspecific nature of the symptoms, their overlap with other conditions, and their presently unclear aetiology lie at the core of the controversy regarding the very existence of the nhSPC syndrome. Yet, an increasing number of neuroscience centres across the world have been offering PC resection as a management option for selected patients with nhSPC, reporting good outcomes.^6^^,^^8^^,^^11^ The evidence behind this practice consists entirely of case reports and retrospective case series and must be interpreted with caution. Recent global survey data mapped the spectrum and scale of the problem and provided insights into the patient perspective—highlighting the current lack of consensus on the management of suspected nhSPC syndrome and the resulting burden to individual patients and healthcare system.^13^

A randomised controlled trial (RCT) is needed to compare efficacy and safety of surgery versus conservative management of nhSPC. However, significant challenges presently hinder its implementation. These include the relative rarity of the syndrome, the paucity of neurosurgical expertise, and barriers to recruitment inherent to RCTs comparing surgery with non-surgical interventions. Thus, evidence from a prospective cohort study is a necessary step prior to an RCT.

Here, we report the results of the first prospective cohort study of surgical management of nhSPC, the Cambridge Symptomatic Pineal Cyst Prospective Cohort Study (CamProS-PC). We aimed to collect prospective evidence on the safety and efficacy of surgical intervention in nhSPC and to determine the feasibility of conducting a definitive RCT.

## Methods

### Study design

#### Registration

CamProS-PC is an investigator-initiated, pragmatic, observational, single-centre prospective cohort study. The study protocol was reviewed by the Health Research Authority (HRA) and Health and Care Research Wales (HCRW) and was approved in July 2021 (21/NI/0120, IRAS292313)—its full version is available in the Appendix (pp. 2–60) and on our institutional page (cctu.org.uk). This study is registered at ISRCTN (ISRCTN51545574).

Although the study was formally launched in July 2021, identical surveys had been collected prospectively from all patients undergoing PC resection using the same eligibility criteria as part of the unit's routine clinical practice since January 2019. The Covid-19 pandemic substantially reduced the rate of recruitment—in order to facilitate the recruitment of a sufficient number of patients to appropriately power this study, on 1/2/2022 the Ethics Committee approved an amendment of the study protocol to include these consecutive patients and their prospectively collected data in the CamProS-PC cohort.

#### Participants

All consecutive patients (N = 122) referred to the Neurosurgical Department of Addenbrooke's Hospital between January 2019 and May 2023 with a PC were screened against eligibility criteria. The last patient (n = 40) was recruited in May 2023, and the last follow-up entry required to capture the primary outcome was obtained in May 2024.

Patients were offered participation in the study if they consented to undergo surgical treatment of a symptomatic PC. The indication for surgery and the study inclusion criteria were identical: (1) age >18 years; (2) presence of a PC of size >10 mm; (3) presence of severe symptoms consistent with the syndrome of nhSPC, defined as ≥6/10 on an established severity scale^6^; (4) minimum of 6 months of conservative treatment without improvement.

In addition, patients were deemed ineligible if they met any of the following exclusion criteria: (1) radiological evidence of ventriculomegaly; (2) other diagnosis of CNS pathology, including neoplasm, vascular (ischemic or haemorrhagic), traumatic, or hydrocephalus; (3) history of intracranial neurosurgical intervention.

Written informed consent was obtained for all patients recruited in the study.

#### Procedures

All eligible participants (N = 40) underwent surgery as per normal clinical pathway. MR imaging was performed before and 12 months after surgery. Cyst size was defined as the greatest diameter of the entire pineal body, including the cyst and any discernible pineal tissue.

Patients were asked to complete the EORTC-QLQ-C30 questionnaire and a study-specific questionnaire in physical or digital format before surgery and at 3 months, 12 months, and annually after surgery.

The EORTC-QLQ-C30 questionnaire is widely used to assess health-related quality of life (HRQoL) of patients across multiple domains.^14^ The RF domain was chosen as the primary outcome of the study because the inability to fulfil their role in family, work, and society has consistently been a primary concern for patients with nhSPC.^13^

The study-specific questionnaire before surgery was designed to survey demographic characteristics, and quality and severity of disease-specific symptoms. In the final section, patients were given free space to list and describe additional symptoms (Appendix pp. 85, 90).

The study-specific questionnaire after surgery was designed to measure the progression of each symptom relative to the preoperative status categorised into six levels: no longer have this symptom, much better, better, no different, worse, much worse (Appendix pp. 83–85, 88–90).

#### Surgical intervention

PC resection was caried out via unilateral paramedian supracerebellar infratentorial approach as previously described, but in the prone position.^15^ All surgeries were performed by a single surgeon (TS). The aim was to resect more than 50% of the wall of the cyst, balancing the risk of recurrence against the risk of injuring the surrounding structures.

#### Complications

Complications were recorded prospectively by the investigating team and via patient questionnaires. They were then classified as temporary or permanent depending on whether they were present at 12 months postoperatively, and according to Clavien-Dindo^16^ and Landriel Ibanez^17^ classification of complications in neurosurgery.

#### Outcomes

The primary objective of the study is improvement by 20 points in the European Organisation for Research and Treatment of Cancer Quality of Life Questionnaire—C30 (EORTC QLQ-C30) Role Functioning (RF) scale score at 12 months postoperatively as compared to preoperatively. This cut-off was chosen based on the published literature suggesting that a 20-point improvement reflects a “medium or large change” in health-related quality of life.^18^, ^19^, ^20^ The sample size N = 40 was determined to power the study to detect this difference with an alpha = 0.05 and beta = 0.10 using estimated standard deviation of 42 at baseline and 18 post-surgery with a conservative assumption of 0.5 correlation as detailed in the study protocol.

Secondary objectives were (1) to characterise the effect of surgery on all other domains of the EORTC-QLQ-C30 questionnaire at 3 months, 12 months and every subsequent year postoperatively compared to the preoperative level; (2) to characterise the cohort of recruited patients with nhSPC and measure the rate of symptom improvement at 3 months, 12 months, and every subsequent year; (3) to assess the safety of the intervention by prospectively collecting complications.

Exploratory objectives aimed to identify radiological correlates of nhSPC. In addition, we interrogated the data looking for biomarkers that could be positively or negatively associated with outcomes, potentially assisting in risk stratification. Predictors considered included demographics, clinical characteristics, and radiological variables.

### Statistical analysis

Before-after analysis of continuous variables was performed via paired Wilcoxon signed-rank test because the assumption of normality of the distributions could was not met according to the Shapiro–Wilk test of normality, adjusting for multiple comparisons when required. For completeness, t-test p-values were also computed and confronted with p-values computed from Wilcoxon signed-rank tests, confirming that in all cases the latter provided a more conservative p-value estimation. Before-after analysis of categorical variables was performed using a Chi-Square or McNemar test for unpaired and paired comparisons, respectively. Analysis of the association between variables and outcomes were performed using univariable and multivariable linear regression if the outcome is continuous (e.g. HRQoL score), or logistic regression if dichotomous. Continuous variables were not dichotomised. All statistical analyses were performed in R. All p values are reported as unadjusted unless specified. Where appropriate, the p = 0.05 was adopted as threshold for statistical significance. Given the low proportion of missing data (≤2.5%), complete-case analysis was performed, and the number of patients included in each analysis is specified. In all cases, patient-level data is provided in the Supplementary Materials.

### Missing data

For one patient the preoperative EORTC-QLQ-C30 questionnaire was not received. For a different patient the 3-month EORTC-QLQ-C30 questionnaire and the study-specific questionnaire were not received. At 12 month, all questionnaires were received. The low proportion of missing data (≤2.5%) is unlikely to introduce bias in the study. In all instances complete-case analysis was performed, and the number of patients included in each analysis is specified. In all cases, patient-level data is provided in the Supplementary Materials.

### Risk of bias

Participant performance bias is intrinsic to the nature of open-label surgical trials, and no mitigation was possible. To reduce the effect of investigator performance bias, data registration of all patients' baseline characteristics was performed by blinding the investigator to the patients’ outcomes. Because CamProS-PC is the first prospective study in the field of nhSPC surgery, it was not ethically nor practically possible to include a control group.

### Role of the funding source

The funder of the study had no role in study design, data collection, data analysis, data interpretation, or writing of the report.

## Results

**Fig. 1:**
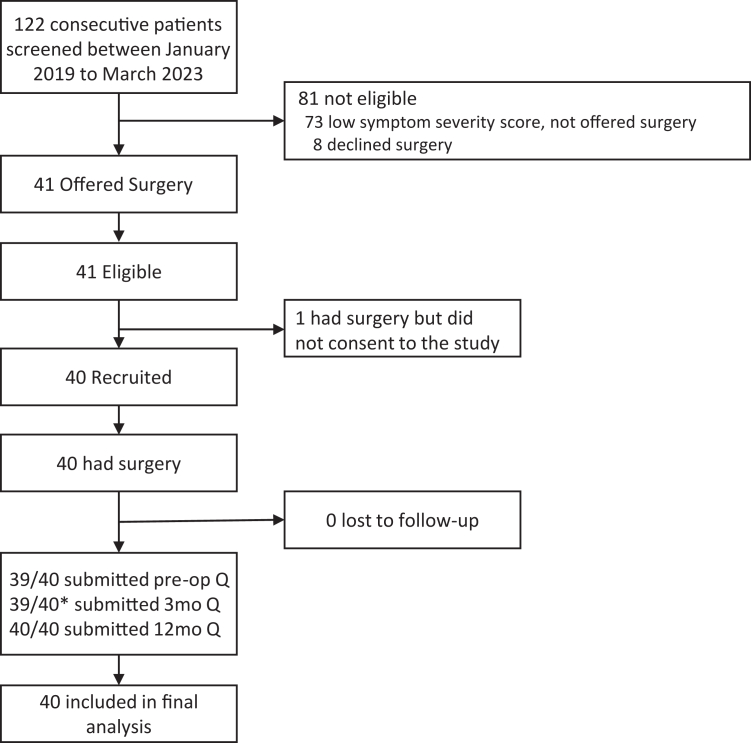
Study profile. ∗The patient who did not submit the pre-op questionnaire is not the same patient who did not submit at 3 months.

Between 1st January 2019 and 31st May 2023, a total of 122 patients with nhSPC were referred to the Neurosurgical Unit at Addenbrooke's Hospital. Of these 41 were eligible according to inclusion and exclusion criteria, and 40 consented to participate in the study. There was no loss of follow-up. Fig. 1 shows the CONSORT diagram of the recruitment process, including the reasons for non-eligibility.

### Cohort characteristics

**Fig. 2:**
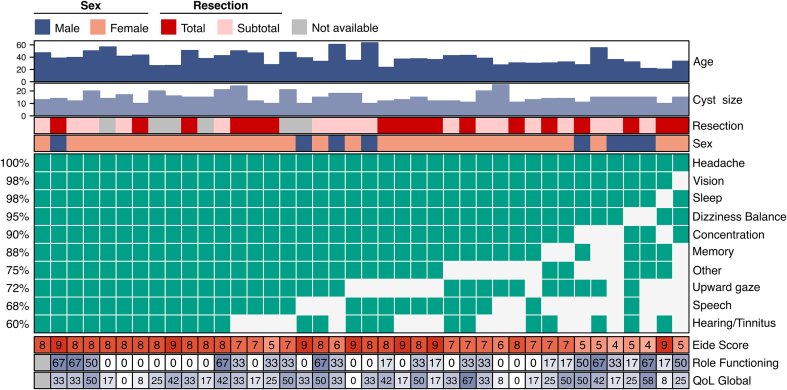
Cohort characteristics. Heatmap summarising the characteristics of the CamProS-PC cohort. The top annotation summarises demographic characteristics: age, cyst size, extent of resection, sex assigned at birth, and surgical approach. The body of the heatmap reports presenting symptoms for each patient. The bottom annotation shows symptom severity, role functioning, and global quality of life scores before surgery, respectively.

A summary of cohort characteristics is provided in Fig. 2 and Table 1.

**Table 1:** Characteristics of the patients in the CamProS-PC cohort.

	Mean (±SD) or Pct (n/N)
**Demographics**	
Age (years)	38 (28–49)
Sex (Female)	80% (32/40)
Full-time Employment	49% (19/39)
**Symptoms**	
Headache	100% (40/40)
Vision	98% (39/40)
Upward Gaze	72% (29/40)
Dizziness/Balance	95% (38/40)
Hearing/Tinnitus	60% (24/40)
Memory	88% (35/40)
Concentration	90% (36/40)
Sleep	98% (39/40)
Speech	68% (27/40)
Other	75% (30/40)
**Radiological**	
Cyst size (mm)	15 (11–19)
Rd (mm)	0.9 (0.4–1.3)
Cd (mm)	1.5 (1.2–1.8)
Rd/Cd Ratio	0.6 (0.3–0.8)
Tectum compression (% patients)	90% (36/40)
Splenium compression (% patients)	43% (17/40)
**Surgical**	
Resection extent (% resection)	87% (71%–100%)
Approach (% SCIT)	100% (40/40)

Rd, Rostral diameter of aqueduct; Cd, Caudal diameter of aqueduct; SCIT, Supracerebellar Infratentorial approach.

The mean age was 38 years (range 20–63), 80% (32/40) were females and 48% (19/40) were in full time employment. The mean cyst size was 15 mm (range 10–29) (Table 1). The mean duration of symptoms was 5 years (range 9 months–31 years). Prior to recruitment, 89% (34/38) reported seeing at least once a neurologist, 58% (21/36) an ophthalmologist, and 31% (12/38) an otolaryngologist (Supplementary Table S1).

All patients reported headaches (40/40). Other commonly reported symptoms at presentation included visual disturbance 98% (39/40), dizziness 95% (38/40), sleep disturbance 98% (39/40) and problems with concentration 90% (36/40), memory 88% (35/40), speech 68% (27/40) and hearing 60% (24/40). Out of 39 patients reporting visual disturbance, 75% (29/39) had limited or uncomfortable upward gaze suggestive of a Parinaud Syndrome-like phenomenon. Patient-level data of presenting symptoms can be found in Supplementary Table S2.

### Missing data

For one patient the preoperative EORTC-QLQ-C30 questionnaire was not received. For a different patient the 3-month EORTC-QLQ-C30 questionnaire and the study-specific questionnaire were not received. At 12 month, all questionnaires were received. Overall, the proportion of missing data was ≤2.5%.

### Outcomes

#### Primary outcome

The mean improvement in RF score at 12 months postoperatively was 46 [SD 11–80, p < 0.0001). There was no loss of follow-up. All patients were eligible for analysis. The primary endpoint for the CamProS-PC study was therefore met.

#### Secondary outcomes

##### HRQoL

**Fig. 3:**
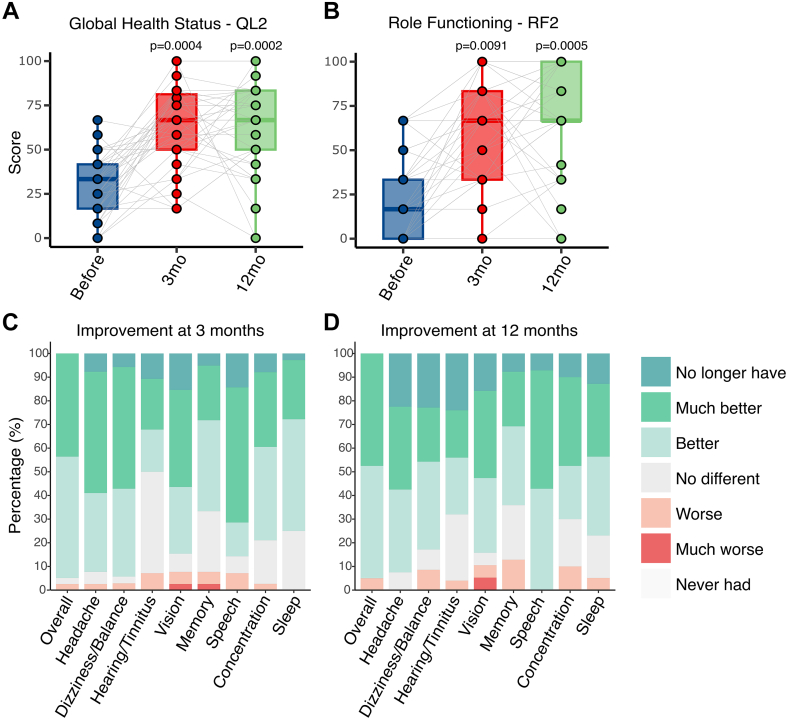
Summary of outcomes. **A, B:** Boxplots of patient-reported quality of life scores for Global Health Status and Role Functioning, respectively, according to the EORTC-QLQ-C30 questionnaire. Identical scores are overlapped. Shaded lines join scores from the same patient. Bonferroni-corrected Wilcoxon p-values are shown relative to pre-op. All other scales are shown in Supplementary Fig. S1. **C, D:** Stacked bar chart of overall and specific symptom outcome as patient-reported at 3 months and at 12 months post-op, respectively.

At 3 months postoperatively, statistically significant improvement was observed across all five functional scales of the EORTC-QLQ-C30 questionnaire: Physical mean difference 17 [SD −9 to 43, p = 0.0006], RF 39 [SD 5–74, p < 0.0001], Emotional 35 [SD 8–62, p < 0.0001], Cognitive 41 [SD 10–73, p < 0.0001], and Social 42 [SD 3–80, p < 0.0001] (Table 2). This improvement was clinically relevant (>20 points) for all scales except Physical Functioning. Global Health Status score improved by 33 points [SD 5–62, p < 0.0001] (Fig. 3). Symptom Scale improvement was observed for fatigue [−37 points (SD −65 to 9, p < 0.0001)], nausea/vomiting [−20 points (SD −48 to 8, p = 0.0062)], pain [−52 points (SD −80 to 24, p < 0.0001)], insomnia [−53 points (SD −89 to 16, p < 0.0001)], and appetite loss [−25 points (SD −56 to 7, p = 0.0034)] (Table 2).

**Table 2:** Summary of outcomes.

	Scale	Before	3 months	12 months	Change at 3 months from baseline		Change at 12 months from baseline	
		Score	Score	Score	Δ Score	p value	Δ Score	p value
**Global health status**								
Global QoL	QL2	30 (13–46)	62 (40–85)	63 (38–87)	33 (5–62)	<0.00001	32 (4–61)	<0.00001
**Functional scales**								
Physical functioning	PF2	59 (33–85)	75 (51–99)	82 (59–104)	17 (−9 to 43)	0.00061	22 (−1 to 46)	<0.00001
Role functioning	RF2	24 (−1 to 48)	63 (34–92)	70 (39–100)	39 (5–74)	<0.00001	46 (11–80)	<0.00001
Emotional functioning	EF	38 (12–63)	73 (46–99)	73 (45–101)	35 (8–62)	<0.00001	35 (−2 to 71)	0.00001
Cognitive functioning	CF	28 (4–52)	70 (46–93)	67 (39–95)	41 (10–73)	<0.00001	38 (3–73)	0.00001
Social functioning	SF	22 (−1 to 45)	62 (34–91)	73 (42–104)	42 (3–80)	<0.00001	50 (14–87)	<0.00001
**Symptom scales**								
Fatigue	FA	82 (62–102)	45 (20–70)	37 (9–66)	−37 (−65 to −9)	<0.00001	−43 (−74 to −13)	<0.00001
Nausea and vomiting	NV	36 (9–63)	17 (−1 to 36)	15 (−9 to 39)	−20 (−48 to 8)	0.00062	−21 (−48 to 6)	0.00024
Pain	PA	90 (76–104)	38 (11–66)	34 (4–64)	−52 (−80 to −24)	<0.00001	−55 (−86 to −24)	<0.00001
Dyspnoea	DY	28 (−3 to 60)	15 (−7 to 36)	15 (−10 to 40)	−13 (−43 to 18)	0.01936	−12 (−44 to 19)	0.02062
Insomnia	SL	86 (61–111)	32 (2–62)	30 (−3 to 62)	−53 (−89 to −16)	<0.00001	−55 (−87 to −23)	<0.00001
Appetite loss	AP	43 (9–76)	18 (−12 to 48)	16 (−12 to 44)	−25 (−56 to 7)	0.00034	−27 (−60 to 7)	0.00009
Constipation	CO	30 (−4 to 64)	15 (−10 to 41)	13 (−18 to 43)	−17 (−48 to 15)	0.00453	−18 (−52 to 16)	0.01510
Diarrhoea	DI	19 (−11 to 48)	10 (−9 to 29)	11 (−14 to 35)	−11 (−43 to 22)	0.04998	−8 (−35 to 19)	0.08222
Financial difficulties	FI	49 (4–94)	32 (0–63)	25 (−8 to 58)	−17 (−63 to 29)	0.01200	−23 (−64 to 18)	0.00130

Quality of life assessment outcomes at 3 months and 12 months according to the EORTC-QLQ-C30 questionnaire. For global health status and functional scales, high scores indicates high quality of life. For symptom scales, high scores indicate high burden of the symptom on quality of life. Scores for individual patients are available as Supplementary Data.

At 12 months, statistically significant and clinically relevant improvement from preoperative baseline persisted across all five functional scales: RF 46 [SD 11–80, p < 0.0001], Physical 22 [SD −1 to 45, p < 0.0001], Emotional 35 [SD −2 to 71.45, p < 0.0001], Cognitive 38 [SD 2.79–73.28, p < 0.0001], Social 50.43 [SD 14.06–86.8, p < 0.0001] (Table 2). Global Health Status improvement persisted [32 points (SD 4–61, p < 0.0001)] (Fig. 3). Similarly, reduced fatigue [−43 points (SD −74 to 13, p < 0.0001)], nausea/vomiting [−21 points (SD −48 to 6, p = 0.00024)], pain [−55 points (SD −86 to 24, p < 0.0001)], insomnia [−55 points (SD −87 to 23, p < 0.0001)] and appetite loss [−27 points (SD −59 to 7, p < 0.0001)] continued to be reported with the addition of reduced financial burden [−23 (SD −64 to 18, p = 0.0013)] (Table 2).

##### Symptom improvement

Overall symptom improvement at 3 and 12 months from preoperative baseline was reported by 95% (37/39) and 95% (38/40) of patients, respectively (Fig. 3). The improvement rate for specific symptoms at 3 months and 12 months, respectively, was 92% (36/39) and 93% (37/40) for headaches, 94% (33/35) and 83% (29/35) for dizziness, 50% (14/28) and 68% (17/25) for hearing/tinnitus, 85% (33/39) and 84% (32/38) for vision, 67% (26/39) and 64% (25/39) for memory, 79% (30/38) and 70% (28/40) for concentration, and 75% (27/36) and 77% (30/39) for sleep (Table 3). At 12 months postoperatively, 5% (2/40) reported feeling overall worse relative to preoperatively. It was not possible to determine whether this was attributed to the surgery or the onset and progression of other concomitant conditions.

**Table 3:** Summary of symptom progression.

	3 months			12 months		
	Improved	Worse	Unchanged	Improved	Worse	Unchanged
Overall	95% (37/39)	3% (1/39)	3% (1/39)	95% (38/40)	5% (2/40)	0% (0/40)
Headache	92% (36/39)	3% (1/39)	5% (2/39)	93% (37/40)	0% (0/40)	8% (3/40)
Dizziness/Balance	94% (33/35)	3% (1/35)	3% (1/35)	83% (29/35)	9% (3/35)	9% (3/35)
Hearing/Tinnitus	50% (14/28)	7% (2/28)	43% (12/28)	68% (17/25)	4% (1/25)	28% (7/25)
Vision	85% (33/39)	8% (3/39)	8% (3/39)	84% (32/38)	11% (4/38)	5% (2/38)
Memory	67% (26/39)	8% (3/39)	26% (10/39)	64% (25/39)	13% (5/39)	23% (9/39)
Concentration	79% (30/38)	3% (1/38)	18% (7/38)	70% (28/40)	10% (4/40)	20% (8/40)
Sleep	75% (27/36)	0% (0/36)	25% (9/36)	77% (30/39)	5% (2/39)	18% (7/39)

Reported symptom progression at 3 months and 12 months post-operatively. Outcomes for individual patients are available as Supplementary Data.

##### Employment

Preoperatively 49% (19/39) of patients reported being in full or part-time employment. This was 41% (16/39) at 3-months follow-up (McNemar's p = 0.44). At 12 months, the employment rate improved to 72% (28/39, McNemar's p = 0.027).

##### Safety

Complications occurred in 23% (9/40). These were classified as per Clavien-Dindo as 4 grade-1, 2 grade-2, 1 grade-3a, 2 grade-3b, or alternatively, using Landriel Ibanez classification as 1 grade-1a, 5 grade-1b, 1 grade-2a, 2 grade-2b (Table 4).

**Table 4:** Summary of complications.

	Incidence (n = 40)
**Overall**	
Any complication	9 (23%)
Temporary (<12 months)	8 (18%)
Permanent (>12 months)	1 (3%)
**Landriel Ibanez classification**	
Grade 1a	1 (3%)
Grade 1b	5 (13%)
Grade 2a	1 (3%)
Grade 2b	2 (5%)
Grade 3	0 (0%)
Grade 4	0 (0%)
**Clavien Dindo Classification**	
Grade 1	4 (10%)
Grade 2	2 (5%)
Grade 3a	1 (3%)
Grade 3b	2 (5%)

Complications classified according to Landriel Ibanez and Clavien Dindo. Data for individual patients are available as Supplementary Data.

There were two cases of wound infection requiring admission for debridement and intravenous antibiotics, two cases of wound infection treated with oral antibiotics in the community, and one case of gastrointestinal bleeding on day three postoperatively that required endoscopy. Two patients reported new visual disturbance: in one this improved to mild at 3 months follow-up and had completely resolved by 12 months; in the other, diplopia persisted and was present at 4 years postoperatively, albeit mild and only noticeable when watching television in a lateral position. The patient scored vision-related symptoms as ‘better’ when compared to pre-operatively. (Table 4 and Supplementary Tables S10 and S11).

Incisional pain was reported by 35% (14/40) of patients at 3 months follow-up and still present, albeit generally milder, in 25% (10/40) at 12 months. All patients were alive at the time of last follow-up.

### Exploratory outcomes

We tested the association between demographic, clinical, and radiological features with disease severity and outcomes. In univariable analysis we found consistent associations between markers of aqueduct compression, such as minimal diameter (Md), rostral diameter (Rd), caudal diameter (Cd), and Rd:Cd ratio, with improved scores on HRQoL domains and symptom outcomes at 12 months (Supplementary Table S23). These remained significant after adjusting for clinical covariates (Supplementary Table S24). For the complete analysis please refer to Supplementary Tables S15–S26.

### Long term follow-up

Among 32 patients with follow-up beyond one year, 31 (97%) returned their questionnaire. The patient who did not was seen in clinic and reported that they are ‘overall better’. The median duration of follow-up for this cohort is 3 years (range 2–5 years). At last follow-up, statistically and clinically significant improvement was observed across all five functional scales: Physical 26 [SD 2–51, p < 0.0001], RF 49 [SD 13–84, p < 0.0001], Emotional 36 [SD 6–66, p < 0.0001], Cognitive 48 [SD 14–81, p < 0.0001], Social 56 [SD 18–93, p < 0.0001]. Global Health Status was improved by a mean 38 points [SD 12–63, p < 0.0001). Overall symptom improvement was reported by 90% (28/31) (Supplementary Tables S5 and S9).

## Discussion

The CamProS-PC is the first prospective study of the surgical management of patients with symptomatic PC without hydrocephalus. At one year following surgery, cyst resection resulted in a mean improvement in RF of 46 points, Global Health Status of 32 points, and overall symptomatic improvement in 95% of patients. The intervention was overall safe, with one long-term neurological complication of visual disturbance and no mortality.

The CamProS-PC has met the primary outcome with no loss of follow-up. Patients reported substantial improvement in RF at one year postoperatively. This was already appreciable at 3 months postoperatively.

In fact, compared to before surgery, patients reported overall statistically and clinically significant improvement in global quality of life and RF at 3 months, and this persisted at 12 months. The burden of all symptoms decreased to a statically significant and clinically relevant extent with the exception of dyspnoea, constipation, and diarrhoea, which have not been previously reported as symptoms of the nhSPC syndrome and were not expected to occur.^5^ (Table 2)

Compared with reference data from healthy controls, nhSPC patients reported considerably worse HRQoL before surgery (mean QL2 30 versus 78 points).^21^ At 12 months postoperatively, HRQoL improved substantially but failed to reach that of healthy controls (mean QL2 63 versus 78), suggesting that although the pineal cyst is a significant aetiological factor it is likely that other factors play a role, either through related or unrelated pathophysiological mechanisms.^21^

RF is a core construct of HRQoL and is defined as the ability of the individual to fulfil responsibilities typical for a specific age and social setting.^22^^,^^23^ In the EORTC QLQ-C30, the RF scale assesses limitations in doing work or other daily activities (item 6) and in pursuing hobbies and other leisure time activities (item 7). Moreover, surgery itself has a potential to substantially impair patient's HRQoL and this must be considered during clinical reasoning and patient counselling.

The prevalence of symptoms in the CamProS-PC cohort was higher than previously reported (Supplementary Table S14).^5^ This is not unexpected, as this is the first prospective collection of patient-reported symptoms of nhSPC syndrome, whereas previous reports were retrospective and surgeon-reported.^5^^,^^6^ Symptomatic improvement following surgery was comparable to that of a meta-analysis of the retrospective literature.^5^

Headache, visual disturbance, and dizziness had the highest improvement rate at 12 months (93%, 84%, 83%, respectively). These are cardinal symptoms of the nhSPC syndrome and putative aetiologies have been proposed relating to tectum compression, altered aqueductal CSF flow and internal cerebral venous insufficiency associated with crowding of the quadrigeminal cistern.^5^, ^6^, ^7^, ^8^, ^9^, ^10^, ^11^

Although lacking direct proof, it is reasonable to hypothesise that vision and balance-associated symptoms such as dizziness, nausea, and unsteady gait may result from interference with tectal processing.^24^, ^25^, ^26^ It is also possible that other manifestations of the nhSPC syndrome, such as cognitive function and fatigue, could be explained by one or more of these mechanisms, especially by the cyst's interference with CSF, venous, and glymphatic flow as demonstrated by Eide et al.^7^^,^^12^ In addition, certain symptoms, especially in patients with predisposing factors, can be a consequence of functional overlay where PC-related symptoms, such as constant headaches, can serve as both precipitating and perpetuating factors compounded by years, sometimes decades, of medically unexplained symptoms that impair the ability to fulfil personal and social roles.^27^

The safety profile of the intervention in this prospective study is comparable with previous reports showing that surgery performed by specialist neurosurgical teams is a safe treatment option for nhSPC syndrome.^5^ Complications are classified according to Clavien-Dindo^16^ and Landriel Ibanez^17^ classifications and results from this cohort can be used as a benchmark for future studies.

We observed consistent associations between markers of aqueduct compression, such as minimal diameter (Md), rostral diameter (Rd), caudal diameter (Cd), and Rd:Cd ratio, and improved aspects of HRQoL and symptom outcomes (Supplementary Tables S15–S24). This finding further supports the hypothesis of nhSPC syndrome arising from mechanistic alteration of the physiology of the quadrigeminal plate and aqueduct secondary to mass effect of the PC.^2^^,^^5^, ^6^, ^7^, ^8^, ^9^, ^10^, ^11^^,^^28^ Further studies with more patients, high-resolution, and novel MRI sequences are under way and will yield additional insight into the aetiology of the nhSPC syndrome.

The beneficial effect of surgery persisted beyond the one-year timepoint prespecified for the primary outcome of the study. At the time of writing this manuscript, statistically significant and clinically relevant improvement was observed across all five functional scales of the EORTC-QLQ-C30 questionnaire, Global Health Status, and overall symptomatic burden, with a median follow-up of 3 years (range 2–5).

This study had some limitations. It was not possible to mitigate patient performance bias and placebo effect, which is intrinsic to non-randomised surgical cohort studies. It is possible that this study has overestimated the effect size of the intervention, especially given the subjective nature of the outcome measures.^29^^,^^30^ To dissect the effect of placebo from the true effect of PC resection, a randomised placebo-controlled trial is required.^31^ This study provides the necessary evidence to justify a placebo-controlled RCT of safety and efficacy of cyst resection to treat nhSPC syndrome.

Generalisability is limited by the pragmatic single-centre and single-surgeon nature of this observational cohort study. Yet, the results are informative and valid for patients with characteristics that are similar to those of this cohort, which can be considered representative of a subset of patients with nhSPC syndrome who are severely symptomatic. Long-term follow-up is required to quantify the duration of the effect of surgical intervention.

Lastly, this study did not collect longitudinal data regarding potential confounders such as changes in BMI, lifestyle behaviour, medications, and analgesic use and therefore all estimates could not be adjusted for these covariates.

In conclusion, CamProS-PC study is the first prospective cohort study to rigorously investigate the efficacy and safety of surgical management of patients with severe nhSPC syndrome. It demonstrated significant benefits to HRQoL and symptomatic burden at 12 months postoperatively, with risks of morbidity and mortality that are comparable to those of previously reported retrospective studies.^5^ It provides evidence to justify a randomised placebo-controlled trial of safety and efficacy of PC resection compared to conservative management in patients with severe symptoms.

## Contributors

TS conceptualised the study. TS, RM, WQ developed the protocol. TS, RM, JH implemented the study. RM, WQ did the statistical analysis. All authors had access to raw data. TS, RM, JH verified the underlying data. TS, RM wrote the first draft of the manuscript. All authors reviewed the analyses and drafts of this manuscript and approved its final version.

## Data sharing statement

Data collected for the study, including patient-level data, are provided in the Supplementary Materials. The study protocol, patient information sheet, and all questionnaires used in the study are available in the appendix.

## Declaration of interests

The authors declare no competing interests.
