# Supplementary material for: Surgery for pineal cysts with symptoms in the absence of hydrocephalus: a prospective cohort study
Source: eClinicalMedicine. 2025 Sep 19;89:103514. doi: 10.1016/j.eclinm.2025.103514 (PMC12495433; doi:10.1016/j.eclinm.2025.103514)
Supplement: Appendix [file mmc1.pdf]

APPENDIX

Contents:

1. Study protocol
2. Study protocol amendment
3. Patient information sheet
4. Literature review and meta-analysis
5. EORTC-QLQ-C30 Questionnaires
6. Study-specific questionnaire – preoperative
7. Study-specific questionnaire – 3-month postoperative
8. Study-specific questionnaire – 12-month postoperative

1. Study protocol

Study Title: Assessment of changes in quality of life and symptoms over time in patients with symptomatic pineal cyst treated with surgery or conservatively - a prospective observational cohort study

IRAS Project ID: 292313

Date and Version No: 28/06/2021 Version 1.2

Chief Investigator:	Thomas Santarius
Investigators:	Anna Hill<br>Peter Hutchinson<br>Alexis Joannides<br>Angelos Kolias<br>Riccardo Masina<br>Wendi Qian<br>Amber Steele
Sponsor:	Cambridge University Hospitals NHS Foundation Trust

1. SYNOPSIS

**Study Title**	Assessment of changes in quality of life and symptoms over time in patients with symptomatic pineal cysts treated with surgery or conservatively - a prospective observational cohort study
**Internal ref. no.**	
**Study Participants**	Patients with symptomatic pineal cysts (SPCs)
**Planned Sample Size**	40 surgically treated patients and all conservatively treated patients at the same period
**Planned Study Period**	Up to 5 years (2-year recruitment, 1 –year follow up, 2 more years for long-term follow up)
**Primary Objective**	Assessment of the impact of surgery on quality of life, specifically patients' role functioning at one year after resection of symptomatic pineal cyst.
**Secondary Objectives**	1. Assessment of all aspects of quality of life as defined by the EORTC QLQ-C30 questionnaire at 1 year and also at 2 and 3 years after surgery.<br>2. Assessment of symptoms at 1 year and also at 2 and 3 years after surgery.<br>3. Assessment of all aspects of quality of life and symptoms in patients who did not undergo surgery (= conservative management) at the time of diagnosis and at 1, 2 and 3 years after joining the study; and investigation the differences at baseline and follow up between those treated surgically and conservatively.<br>4. Comparison of objective neuropsychological assessments before the operation and at 1 year after surgery.

	5. Development of a model to identify anatomical (based in MRI scans) and clinical (symptoms) predictors of improvement of QoL after surgery for SPC
**Study design**	Prospective observational cohort study
**Interventions**	No interventions. This is an observational study.

2. BACKGROUND AND RATIONALE

Pineal cysts (PCs) are benign, non-cancerous cysts arising from the pineal gland. While PCs are common (they can be identified on 2-4% of MRI scans(Golzarian *et al.*, 1993)(Mamourian and Towfighi, 1986)(Nevins *et al.*, 2016)), symptomatic pineal cysts (SPCs) are much rarer. While incidental PCs (i.e. discovered incidentally on a scan done for another reason) are thought to have no long-term consequences, patients with SPCs may suffer a number of chronic and acute symptoms, including headaches, vision and eye movement abnormalities, impairment of balance problems, speech and other cognitive problems, hydrocephalus, pineal apoplexy and even sudden death as a direct result of their pineal cyst (Nevins *et al.*, 2016)(Koziarski, Podgórski and Zieliński, 2019)(Májovský, Netuka and Beneš, 2017)(Fedorko, Zweckberger and Unterberg, 2018)(Kalani *et al.*, 2015)(Eide and Ringstad, 2017)(Choque-Velasquez *et al.*, 2019)(El Damaty *et al.*, 2019).

The exact incidence of SPCs is not known but, based on the numbers of contacts made with Pineal Cyst UK (PCUK) and the number of new patients we saw in the Eastern Region in 2019, we estimate that there are 200 new cases of SPC in the UK each year. The average age of patients with SPC is 35 years and the symptoms have major impact on patients' quality of life (QoL), including their role in family, work and society at large(Fedorko, Zweckberger and Unterberg, 2018).

Joanne Warren, an Australian SPC patient and a patient group moderator, has shared with us a survey of people with SPCs (79%) and pineal tumours (21%) the group conducted in 2018(Joanne Warren, 2018). The most frequently reported symptoms were headaches, visual symptoms, fatigue, sleep problems, hearing and balance problems, feeling of being disconnected from the surroundings, speech, memory and other cognitive problems. Very similar lists of symptoms have also been reported in the medical literature(Májovský, Netuka and Beneš, 2017)(Májovský, Netuka and Beneš, 2018)(Májovský, Netuka and Beneš, 2017)(Májovský, Netuka and Beneš, 2016)(Koziarski, Podgórski and Zieliński, 2019)(El Damaty *et al.*, 2019)(Kalani *et al.*, 2015)(Fedorko, Zweckberger and Unterberg, 2018)(Choque-

Velasquez *et al.*, 2019). These symptoms have a significant impact on patients' QoL and this has also been shown in the literature(Májovský, Netuka and Beneš, 2017)(Fedorko, Zweckberger and Unterberg, 2018). The most detrimental impact was recorded in "role functioning" domains, i.e. inability to fulfil roles in family, work and society at large. It has been reported that due to disability attributed to the SPCs, two thirds of patients were unable to carry out their normal activities and stay in work(Eide and Ringstad, 2017) (Fedorko, Zweckberger and Unterberg, 2018). Until recently, pineal cysts have rarely been considered to be the cause of patients' symptoms, unless causing hydrocephalus and/or Parinaud syndrome.

In the last five years eighth case series showing significant improvement in the symptoms and QoL after resection of SPCs have been published (Table 1). In these papers, improvement is reported in over 90% patients(Májovský, Netuka and Beneš, 2017)(Kalani *et al.*, 2015)(El Damaty *et al.*, 2019)(Koziarski, Podgórski and Zieliński, 2019)(Fedorko, Zweckberger and Unterberg, 2018)(Choque-Velasquez *et al.*, 2019)(Eide and Ringstad, 2017)(Choque-Velasquez *et al.*, 2019). Fedorko et al assessed QoL in a cohort of 12 patients with SPCs, which demonstrated a three-fold increase in the ability to carry out daily activities following surgery (Fedorko, Zweckberger and Unterberg, 2018). In another report, two thirds of patients who were unable to work prior, returned to work following surgery(Eide and Ringstad, 2017).

These studies showed that surgery can bring life-changing benefits to patients with SPC, signalling a potential paradigm shift in thinking about pineal cysts. However, given the relative novelty of these findings, the lack of understanding of the mechanism by which PC can cause symptoms as well as frequent co-existence with other medical conditions SPCs are frequently misdiagnosed and mistreated. In the cited survey by Warren(Joanne Warren, 2018) the majority of the responders had been assessed for and diagnosed with a variety of conditions, including migraine, cluster headaches, chronic fatigue syndrome, anxiety and depression. Not infrequently, when symptomatic management fails to bring improvement, the symptoms are labelled as "functional", i.e. having no material basis. This further compounds patients'

feelings of helplessness, isolation and dejection. Often, several layers of symptoms can accumulate, making the task of identifying the true root cause very difficult.

It is therefore essential to learn more about the safety and efficacy of surgery in controlling patients' symptoms and its effect on patients' quality of life, and to determine the predictive value of symptoms, radiological findings and their combination on patients quality of life after surgery. We propose a prospective observational cohort study that will prospectively collect clinical and radiological data on all patients with SPCs, whether they chose surgically or non-surgical (conservative) management. This will provide higher level of evidence regarding the safety and efficacy of improving patients' QoL than the published retrospective studies, but will also provide valuable information for predicting such improvement based on prospectively and systematically collected clinical and radiological data. Ultimately, a randomised control trial of surgery versus conservative management is likely to be required to provide definitive evidence for the efficacy of surgery and this prospective study will serve as a pilot for planning of such an RTC.

Year	Journal	1st author	Country	N	Symptom improvement*	Improvement in QoL
2015	J of Neurosurgery	Kalani	Australia	18	94%	NFI
2017	World Neurosurgery	Majovsky	Czech Republic	21	95%	NFI
2017	Acta Neurochirugica	Eide	Norway	15	100%	NFI
2019	Surgical Neurology International	Choque-Velasquez	Finland	60	100%	NFI
2019	Brit J Neurosurg	Koziarski	Poland	28	97%	NFI
2019	World Neurosurgery	El Dalmaty	Germany	43	95%	NFI
2019	J of Neurosurgery	Fedorko	Germany	12	100%	Yes
2019	Surg Neurol Internat	Choque-Velasquez	Finland	60	98%	NFI

Table 1. *Symptom improvement - % patients with symptoms improvement after surgery when compare to before surgery

NFI – not formally investigated, QoL – QoL

3. OBJECTIVES

3.1. PRIMARY OBJECTIVE

To assess the impact of surgery on quality of life, specifically patients' role functioning derived by the EORTC QLQ-C30 at one year after resection of symptomatic pineal cyst. Role functioning reflects activities in daily life, which is a higher-order outcome than symptoms, i.e. both symptoms and treatment have an impact on the level of functioning, a clear picture of the functioning of the patient. Role functioning was also the most relevant issue to patients seen in the clinic and the members of Pineal Cyst UK.

3.2. SECONDARY OBJECTIVES

3.2.1. Quality of Life – before and after surgery

We will determine the probability of improvement of overall QoL as well as each individual domain, as defined by the EORTC QLQ-C30 questionnaire (Appendix 1). This will be done by comparing pre-operative and post-operative assessments at 3, 12, 24 and 36 months following resection of SPC.

3.2.2. Symptoms – before and after surgery

We will determine the relative predictive values of individual symptoms and signs as well as their combinations in predicting improvement following resection of SPC. This will be done

by comparing pre-operative and post-operative assessments at 3, 12, 24 and 36 months following resection of SPC.

3.2.3. Quality of life and symptoms of patients treated conservatively

We will assess Quality of Life and Symptoms of patient who chose conservative management at enrolment and at 3, 12, 24 and 36 months following the diagnosis of SPC. These data will also serve to compare baseline Quality of Life and Symptoms between patients managed surgically and conservatively. We will explore the differences of outcomes of patients who choose conservative and surgical treatments with adjusting confounding factors. This cohort will also give us an idea about changes in severity of symptoms and QoL over time.

3.2.4. Cognitive status and emotional wellbeing – before and after surgery

As with most other resective brain surgery, SPC patients undergo detailed objective neuropsychological assessments before and after surgery. For the sake of the study this will be done systematically, with a standard protocol and in predefined intervals, i.e. before and 12 months after surgery. (Appendices 1-3)

3.3. EXPLORATORY SECONDARY OBJECTIVES

3.3.1. MRI-based anatomical and physiological predictors of outcome after surgery

The mechanisms underlying clinical presentation in patients with SPCs is largely unknown. This prospective cohort study offers a unique opportunity to collect data that will help our understanding of the physiological bases of patients' symptoms. We will use MR imaging to understand aetiology of symptoms of in patients with SPCs and to predict improvement of symptoms following surgery. In addition to searching for biomarkers, the data will be used in this exploratory part of the study. We will use MR imaging acquired for routine clinical reasons to determine anatomical (size, deformation of the tectum, relationship with great veins, relationship of size and tecto-callosal distance etc) and physiological (flow through the aqueduct of Sylvius, the deep cerebral venous system) predictors of improvement following resection of SPCs by comparing pre- and post-resection data.

3.3.2. Refinement of indications for surgical treatment of patients with SPCs

Patient clinical characteristics, symptoms and imaging data will be used in a model to predict symptom and QoL improvement following resection of SPCs.

4. PROJECT PLAN

We propose a single-centre, prospective controlled cohort study. Unlike previously published studies, which were retrospective cohort studies, this cohort will have *a priori* defined enrolment criteria, data points, outcomes and is powered to answer the study's questions. Given the patient numbers required (N=40) it is feasible to carry out the study in a single centre. The main advantages this design are maximum homogeneity of the study's variables and simplicity of its execution. However, if there is interest from other UK untints the study will be expanded under strict conditions of adherence to the study protocol and subject to governance approvals. This would accelerate recruitment.

4.1. INCLUSION CRITERIA

- Age =/> 16 years
- Pineal cyst > 9mm in maximum diameter (Májovský et al., 2017)
- Symptom category B (total score ≥5) according to Eide and Ringstad(Eide and Ringstad, 2017) – Figure 1_Eide symptoms
- Willingness and ability to comply with study specific procedures and completion of QoL and symptoms questionnaires.
- Written informed consent

Acta Neurochir (2017) 159:349–361

Table 1 A scale for grading of symptoms in patients with pineal cysts

Symptom	Severity	Score
Headache	No	0
	Minor-moderate	1
	Severe	2
Nausea/vomiting	No	0
	Yes	1
Dizziness	No	0
	Yes	1
Visual disturbances	No	0
	Yes	1
Episodic loss of consciousness	No	0
	Yes	1
Lethargy/fatigue	None-moderate	0
	Severe	1
Cognitive impairment	No	0
	Minor-moderate	1
	Severe	2
Transient neurological deficits	No	0
	Yes	1
Total score		0/10 – 10/10

Symptom category A (none-moderate), 0–4/10 scores; symptom category B (much-severe), 5–10/10 scores

4.2. EXCLUSION CRITERIA

- Evidence for alternative diagnoses/pathological processes causing presenting symptoms

4.3. RECRUITMENT STRATEGY AND SCREENING

When patient receive a diagnosis of a symptomatic pineal cyst (SPC) during a routine neurosurgical clinic consultation at the Addenbrooke's Hospital in Cambridge they will be informed about the possibility of taking part in this study by the patient clinical care team.

Patients referred to neurosurgical clinic for evaluation of their pineal cyst, either symptomatic or asymptomatic will be seen as per current practice. If a diagnosis of a symptomatic pineal cyst (SPC) is made (SPC > 9mm, Eide and Ringstad symptom B category), patients will be screened against inclusion and exclusion criteria (see Section 4.1 and 4.2). If fulfilling the entry criteria, patients will be offered participation in the study. Patients will be advised about the aims, expected benefits and risks of surgery as per current practice. Patients who request surgery will undergo an operation as per existing care pathway. Patients who chose not to

undergo surgery will pursue conservative management as per existing care pathway. Interested patients regardless the pathway they have chosen will be explained the study and will be given Participant Information Sheet (PIS). If they wish to join the study they will sign a Consent Form for participation in the study.

We anticipate that recruitment of the required number of patients treated by surgery will take approximately two years (N=40). The follow up for the primary outcome measure will take additional year. The study specific follow up assessments will be performed at one year after surgery for the primary efficacy outcome measure and SPC-related symptoms review. Given the relatively low burden posed by administering and filling in of the of the questionnaires, one early (3 months following surgery) and two late (at 2 and 3 years after surgery) surveys of symptom control and QoL will be performed (see also attached Flow Chart – Appendix 7 and Flow Table – Appendix 8).

Eligible participants who chose not to undergo surgery (this is an entirely clinical and not a research-related decision) will be recruited the non-surgery (conservative treatment) cohort (see also attached Flow Chart – Appendix 7 and Flow Table – Appendix 8). This is to investigate/explore the QoL difference between surgery and non-surgery patients, both at diagnosis (baseline) and 3, 12, 24 and 36 months after treatment (one of the secondary objectives).

4. 4. NUMBERS AND METHODS OF ANALYSIS.

4.4.1. Primary outcome and sample size

The primary efficacy outcome measure is the change of role functioning scale of the EORTC QLQ c30 questionnaire at 12 months after surgery.

It is anticipated that a considerable clinical effect will be achieved, that is, an improvement of at least 20 points in a 0-100 scale in the role functioning scale. Using the estimated standard deviation of 42 at pre-surgery (retrospectively assessed) and 18 at 6 months post-surgery (prospectively assessed)(Fedorko, Zweckberger and Unterberg, 2018), with a conservative

assumption of the correlation coefficient between pre- and post-surgery scores being 0.5, the estimated standard deviation of the difference between pre and post-surgery is 36. With a 5% significance level, 90% power, 37 participants are required to detect the 20 points using a paired t-test.

Allowing for some non-compliance and to maximise the yield from the secondary outcome measures we aim to recruit 40 participants. The assumptions used for sample size estimation will be monitored by the Study Steering Committee (see below). The conservatively treated patients, i.e. those who chose not to undergo surgery, will be recruited until the completion of surgically-treated patients. No power calculations are required as outcome measures related to the conservatively treated (non-surgery) cohort are among secondary objectives of this study.

4.4.2. Secondary outcomes

The secondary outcomes are:

1. Change in all in items of QoL as defined by the EORTC QLQ-C30 questionnaire before and at 3, 12, 24 and 36 months following resection of SPC

2. Change of individual symptoms before and at 3, 12, 24 and 36 months following resection of SPC

3. Change in the results neuropsychological tests at 12 months following surgery when compared to baseline (before surgery)

4. Difference in severity of symptoms and QoL at baseline between patients who chose surgical and non-surgical treatment

5. Assessment of symptoms and QoL over (at diagnosis and 3, 12, 24 and 36 months later) in patients who chose not to undergo surgery

6. Development of a model to identify anatomical (based in MRI scans) and clinical (symptoms) predictors of improvement of QoL after surgery for SPC

4.4.3. Exploratory outcomes

- development of a model to identify anatomical (MRI) and clinical (symptoms) predictors of improvement of QoL after surgery for SPC

4.4.4. Statistical analyses

The paired t-test will be applied to the primary efficacy analysis for all patients received surgery treatment. A detailed statistical analysis plan will be drafted prior to any analyses performed.

4.5. STUDY PROCEDURES

Individual patient participation – overview (see also Flow Chart and Flow Table):

4.5.1. Screening

a. Patients referred to neurosurgical clinic that are diagnosed with symptomatic pineal cyst (SPC) will be screened against inclusion and exclusion criteria. The treatment will be offered and carried out as per current evidence-based clinical practise. Whether patients chose surgery or conservative management, they will be eligible to be enrolled in the study.
b. Information about the study will be provided to eligible patients by a member of the research team after the end of the first neurosurgery clinic appointment. This can occur face-to-face or over the telephone.
c. Informed consent to participate in the study will be obtained by a member of the research team after the patients have considered the relevant information

about participation. Online completion of consent forms will be considered, in keeping with the reduction of face-to-face appointments due to the pandemic. Paper consent forms will also be available.

4.5.2. Symptoms and QoL questionnaires (see Appendices 1-4)

a. Patients who chose surgical treatment

Study participants will undergo surgery as per normal clinical pathway. The participants will be asked to fill in symptom and quality of life (QoL) questionnaires at the entry to the study (at diagnosis) and at 3, 12, 26 and 36 months after surgery. The participants will be given the options of filling the questionnaire on the paper or on-line.

b. Patients who chose conservative management

The participants will be asked to fill in symptom and quality of life (QoL) questionnaires at the entry to the study (at diagnosis) and at 3, 12, 26 and 36 months after the diagnosis. The participants will be given the options of filling the questionnaire on the paper or on-line.

Once enrolled patients will participate in the study for 3 years, although each participant will be able to leave the study at any time without giving a reason. This will in no way affect their clinical management. This includes the option for patients to choose to undergo surgery at any time even if they have initially elected to pursue conservative management.

4.6. INFORMED CONSENT

Informed consent will be taken from patients in a neurosurgical clinic after the diagnosis of a symptomatic pineal cyst is made, patient expresses interest in taking part in the study and passes through the inclusion and exclusion criteria as eligible for participation in the study.

A member of the research team will take informed consent. The person taking informed consent will be experienced in assessing capacity and aware of the ethical issues involved.

Participants will be provided with the current version of the REC-approved Patient Information Sheet for review.

Participants will be given the opportunity to ask any questions and only once all of their queries have been answered will the consent be taken by the Principal Investigator or suitably qualified, delegated member of the clinical care team. Patients will be informed that they can withdraw their consent at any time without giving reasons for doing so.

4.7. STUDY ASSESSMENTS

Assessment methods:

4.7.1. Questionnaires. – see above

4.8. DEFINITION OF END OF STUDY

The study will begin with at a given start date and will conclude after the last patient completes three-year questionnaire. (see also Flow Chart and Flow Table in the Appendix 7 and 8, respectively):

5. INTERVENTIONS

The only interventions in this study are questionnaires that the patients will be asked to fill in. Please see section 4.6.

6. SAFETY REPORTING

6.1 Safety data collection

This is an observational study where the only interventions are questionnaires and collection of clinical data. Nevertheless, this study presents an opportunity to systematically monitor surgical complications whose governance will fall into the realm of clinical rather than research governance. As such, we don't anticipate research-related events. Nevertheless, we will have system in place whereby any relevant events will feed into existing departmental governance structure.

7. STUDY COMMITTEES

Study Management Group

The Chief Investigator (CI) together with the research team are responsible for the day-to-day running of the study as detailed in study-specific procedures. They will work together as the Study Management Group and will meet monthly.

Study Steering Committee

It will consist of one independent neurosurgeon as the chair, one patient representative and one member of Study Management Group. The Study Steering Committee will monitor the conduct and progress of the study, including recruitment, data completeness and deviations from the protocol. The Study Steering Committee will meet every 6 months while participants are on treatment.

1. ETHICAL & REGULATORY CONSIDERATIONS

Consent

The Informed Consent form must be approved by the REC and must be in compliance with GCP, local regulatory requirements and legal requirements. The investigator must ensure that each study participant is fully informed about the nature and objectives of the study and possible risks associated with their participation.

The investigator will obtain written informed consent from each patient before any study-specific activity is performed. The informed consent form used for this study and any change made during the course of this study, must be prospectively approved by the REC. The investigator will retain the original of each patient's signed informed consent form.

Should a patient require a verbal translation of the study documentation by a locally approved interpreter/translator, it is the responsibility of the individual investigator to use locally approved translators.

Ethical committee review

Before the start of the study or implementation of any amendment we will obtain approval of the study protocol, protocol amendments, informed consent forms and other relevant documents (e.g. advertisements and GP information letters, if applicable) from the REC. All correspondence with the REC will be retained in the Study Master File/Investigator Site File.

Annual reports will be submitted to the REC in accordance with national requirements. It is the Chief Investigator's responsibility to produce the annual reports as required.

Study protocol amendments

Protocol amendments must be reviewed and agreement received from the Sponsor for all proposed amendments prior to submission to the REC.

The only circumstance in which an amendment may be initiated prior to REC approval is where the change is necessary to eliminate apparent, immediate risks to the patients (Urgent Safety Measures). In this case, accrual of new patients will be halted until the REC approval

has been obtained.

Declaration of Helsinki and Good Clinical Practice

The study will be performed in accordance with the spirit and the letter of the declaration of Helsinki, the conditions and principles of Good Clinical Practice, the protocol and applicable local regulatory requirements and laws.

GCP training

All study staff must hold evidence of appropriate GCP training or undergo GCP training prior to undertaking any responsibilities on this study. This training should be updated every 2 years or in accordance with your Trust's policy.

7. ETHICS

7.1. Participant Confidentiality

The study staff will ensure that the participants' anonymity is maintained. Each participant will be allocated a unique study number and will be identified only by this number. The information linking each number and the details of each participant will be stored securely in a separate file. All data will be identified by the participant number only. All documents will be stored securely and only accessible by study staff and authorised personnel. The study will comply with the Data Protection Act which requires data to be anonymised as soon as it is practical to do so.

7.2. Other Ethical Considerations

Recruitment will be performed by a consultant neurosurgeon experienced in performing pineal region surgery. There will be no therapeutic promises, coercion or inducement of any sort.

A minimum of personal data and no participant-identifiable data will be gathered.

The only interventions are questionnaires and most questions are expansions of existing non-standardised symptom and lifestyle clinical interview caried in routine clinical practise.

8. DATA HANDLING AND RECORD KEEPING

All patient data will be anonymised. The participants will be identified by a study specific participant number. The information linking each number and the details of each participant will be stored securely in a separate file. All data will be identified by the participant number only. All data will be stored securely on Cambridge University Hospitals (CUH) NHS Trust computing clusters only. This is secure access and backed up automatically. No data will be stored on personal computers.

9. SPONSORSHIP, FINANCING AND INSURANCE

The study is jointly sponsored by the Cambridge University Hospitals NHS Foundation Trust.

All procedures will be done in NHS premises as part of the routine clinical care for patients.

Financing: No funding is required for this study as the amount of 'research-related' work is minimal and this work will be done on volunteer bases by the study participants and investigators.

10. POTENTIAL EXPANSIONS OF THE STUDY

This study may be expanded to a small number (one or two) of additional UK-based neurosurgical centres. Amendments to protocols and ethics application will be submitted as required prior to conducting any such potential expansions.

11. THE TEAM

The Team of experienced academic clinicians, scientists and a patient representative has been assembled to develop and execute a study.

Investigators

Miss Anna Hill, Patient Representative, Pineal Cyst UK

Prof Peter Hutchinson, BSc MBBS PhD FRCS(SN) – Consultant Neurosurgeon, University of Cambridge, Cambridge University Hospitals NHS Trust

Mr Alexis Joannides, MA PhD MB BChir FRCS(SN) – Consultant Neurosurgeon, University of Cambridge, Cambridge University Hospitals NHS Trust

Mr Angelos Kolias, MD MSc PhD FRCS(SN) – Clinical Lecturer, University of Cambridge, Cambridge University Hospitals NHS Trust

Riccardo Mesina, BSc (Hons) – Medical Student, University of Cambridge

Dr Wendi Qian, PhD – Senior Statistician, Cambridge Clinical Trials Unit, University of Cambridge

Dr Amber Steele, PhD – Research Advisor, Cambridge Research Design Services, National Institute for Health Research

Collaborators

Dr Timothy Ham, MB BS BSc PhD – Consultant Neurologist, Cambridge University Hospitals

Prof Manohar Bance – Professor of Otolaryngology, Consultant ENT Surgeon, University of Cambridge. Internationally recognised expert and leading researcher in the field of hearing and balance. Hearing and balance are among the important symptoms.

Many patients present with vertigo and hearing problems and these patients are often referred to ENT specialists. To assure highest quality of relevant clinical data, rigorous and systematic evaluation of each patient by an ENT surgeon with interest in hearing and balance is vital.

Prof Marek Czosnyka, DSc, PhD - Brain Physics, University of Cambridge. Internationally recognised leader in the field of brain physics, especially in cerebrospinal fluid (CSF) dynamics and intracranial pressure (ICP) monitoring and analysis. Discovered and described a number of fundamental principles of CSF and ICP physiology, their measurement and use in the clinical practise. MC's team expertise will be useful in trying to understand the pathogenesis of symptoms in SPCs.

Dr Linda Dirven, PhD – Senior Researcher, Leiden University Medical Center, The Netherlands. Linda Dirven is an internationally recognised leader in the measurement of outcome in neurological and neurosurgical conditions. Developed and validated tools to measure symptoms and quality of life (QoL). LD advised on selection of the most relevant tools to measure changes in quality of life and symptoms following surgery in this patient population.

Dr Tomasz Matys, MD PhD, academic neuro-radiologist with special interest in MR imaging of brain tumours, neuro-degenerative conditions and cerebrospinal fluid.

TM's expertise has been used in the selection of relevant imaging sequences and TM will lead analysis and interpretation of imaging data.

Miss Brinda Muthusamy, MBChB, MRCP UK, FRCOphth Neurosciences, Ophthalmology Consultant Neuro-ophthalmologist with extensive clinical experience in conditions related to neurosurgical practise, including brainstem and pineal region pathology. Has evaluated close to 100 patients with symptomatic pineal cysts (most referred by the Lead Investigator). Visual symptoms are the second most common symptoms in patients with pineal cysts and neuroophthalmological expertise is vital to detect and record this appropriately.

Neuropsychology team led by Dr Emma Woodberry with participation by Dr Alexa McDonald, Dr Priya Varma. The neuropsychology team is recognised by the CQC as providing an 'outstanding' service to patients. The team works closely with the brain tumour team, routinely evaluating patients before and after surgery. The team has the expertise and structure to create a comprehensive and objective snapshot of the neuropsychological status of patients including their cognitive functioning and

emotional wellbeing. As patients with SPCs frequently develop neuropsychological symptoms, it is vital to record these and compare the pre-operative and postoperative status.

13. REFERENCES

Choque-Velasquez, J. *et al.* (2019) 'The microsurgical management of benign pineal cysts: Helsinki experience in 60 cases', *Surgical Neurology International*, 10, p. 103. doi: 10.25259/SNI-180-2019.

Eide, P. K. and Ringstad, G. (2017) 'Results of surgery in symptomatic non-hydrocephalic pineal cysts: role of magnetic resonance imaging biomarkers indicative of central venous hypertension.', *Acta neurochirurgica*, 159(2), pp. 349–361. doi: 10.1007/s00701-016-3029-4.

El Damaty, A. *et al.* (2019) 'Pineal Cyst without Hydrocephalus: Clinical Presentation and Postoperative Clinical Course After Infratentorial Supracerebellar Resection', *World Neurosurgery*, 129, pp. e530–e537. doi: 10.1016/j.wneu.2019.05.200.

Fedorko, S., Zweckberger, K. and Unterberg, A. W. (2018) 'Quality of life following surgical treatment of lesions within the pineal region', *Journal of Neurosurgery*, pp. 1–10. doi: 10.3171/2017.7.JNS17260.

Golzarian, J. *et al.* (1993) 'Pineal cyst: normal or pathological?', *Neuroradiology*, 35(4), pp. 251–253. doi: 10.1007/bf00602604.

Joanne Warren (2018) 'Pineal Cysts and Tumours Independent Survey of Symptoms 2018'.

Kalani, M. Y. S. *et al.* (2015) 'Pineal cyst resection in the absence of ventriculomegaly or Parinaud's syndrome: clinical outcomes and implications for patient selection', *Journal of Neurosurgery*, 123(2), pp. 352–356. doi: 10.3171/2014.9.JNS141081.

Koziarski, A., Podgórski, A. and Zieliński, G. M. (2019) 'Surgical treatment of pineal cysts in non-hydrocephalic and neurologically intact patients: selection of surgical candidates and clinical outcome', *British Journal of Neurosurgery*, 33(1), pp. 37–42. doi: 10.1080/02688697.2018.1530731.

Májovský, M., Netuka, D. and Beneš, V. (2016) 'Clinical management of pineal cysts: a worldwide online survey.', *Acta neurochirurgica*, 158(4), pp. 663–669. doi: 10.1007/s00701-016-2726-3.

Májovský, M., Netuka, D. and Beneš, V. (2017) 'Conservative and surgical treatment of patients with pineal cysts: A prospective case series of 110 patients.', *World neurosurgery*, 105, pp. 199–205. doi: 10.1016/j.wneu.2017.05.155.

Májovský, M., Netuka, D. and Beneš, V. (2018) 'Is surgery for pineal cysts safe and effective? Short review', *Neurosurgical Review*, 41(1), pp. 119–124. doi: 10.1007/s10143-017-0876-2.

Mamourian, A. C. and Towfighi, J. (1986) 'Pineal cysts: MR imaging', *AJNR. American journal of neuroradiology*, 7(6), pp. 1081–1086.

Nevins, E. J. *et al*. (2016) 'Incidental Pineal Cysts: Is Surveillance Necessary?', *World neurosurgery*, 90, pp. 96–102. doi: 10.1016/j.wneu.2016.02.092.

2. Study protocol amendment

Study Title: Assessment of changes in quality of life and symptoms over time in patients with symptomatic pineal cyst treated with surgery or conservatively - a prospective observational cohort study

IRAS Project ID: 292313

Date and Version No: 28/06/2021 Version 1.2

Amended: Amendment 1 - 1/2/2022

Chief Investigator:	Thomas Santarius
Investigators:	Anna Hill<br>Peter Hutchinson<br>Alexis Joannides<br>Angelos Kolias<br>Riccardo Masina<br>Wendi Qian<br>Amber Steele
Sponsor:	Cambridge University Hospitals NHS Foundation Trust

1. SYNOPSIS

**Study Title**	Assessment of changes in quality of life and symptoms over time in patients with symptomatic pineal cysts treated with surgery or conservatively - a prospective observational cohort study
**Internal ref. no.**	
**Study Participants**	Patients with symptomatic pineal cysts (SPCs)
**Planned Sample Size**	40 surgically treated patients and all conservatively treated patients at the same period.<br><br>As a result of Amendment 1, 21 consecutive patients who underwent an operation during the 5-year period prior to the commencement of the study (i.e. since 1st January 2016) and completed the required questionnaires and consented to participating in the study will be included in the total number of 40 surgically treated patients. These patients will be included in the total number of 40 surgically treated patients.<br><br>The primary outcome will be assessed once 40 surgically treated patients reach 12-months follow up. Since all the data required to assess the primary outcome was collected as a part of clinical care and will be available if patients consent for this to be used in the study, it may only be necessary to recruit 19 patients since the commencement of the study in order to achieve the primary objective as well as secondary objectives 3.2.1. and 3.2.2. of the study.<br><br>We will analyse and publish the result of the study with N=40. The cohort will consist of patients operated on before and after the commencement

	of the study. We will also evaluate the available data pertaining to the rest of the secondary objectives (3.2.3, 3.2.4) and exploratory objectives (3.3.1,3.3.2). These data will be less complete and results less informative as a consequence of the Amendment, but the steering committee believe this is a practically and ethically beneficial compromise to mitigate the negative impact of the covid-19 pandemic.
**Planned Study Period**	Up to 5 years (2-year recruitment, 1 –year follow up, 2 more years for long-term follow up)
**Primary Objective**	Assessment of the impact of surgery on quality of life, specifically patients' role functioning at one year after resection of symptomatic pineal cyst.
**Secondary Objectives**	1. Assessment of all aspects of quality of life as defined by the EORTC QLQ-C30 questionnaire at 1 year and also at 2 and 3 years after surgery.<br>2. Assessment of symptoms at 1 year and also at 2 and 3 years after surgery.<br>3. Assessment of all aspects of quality of life and symptoms in patients who did not undergo surgery (= conservative management) at the time of diagnosis and at 1, 2 and 3 years after joining the study; and investigation the differences at baseline and follow up between those treated surgically and conservatively.<br>4. Comparison of objective neuropsychological assessments before the operation and at 1 year after surgery.<br>5. Development of a model to identify anatomical (based in MRI scans) and clinical (symptoms) predictors of improvement of QoL after surgery for SPC
**Study design**	Prospective observational cohort study

**Interventions**	No interventions. This is an observational study.

2. BACKGROUND AND RATIONALE

Pineal cysts (PCs) are benign, non-cancerous cysts arising from the pineal gland. While PCs are common (they can be identified on 2-4% of MRI scans(Golzarian *et al.*, 1993)(Mamourian and Towfighi, 1986)(Nevins *et al.*, 2016)), symptomatic pineal cysts (SPCs) are much rarer. While incidental PCs (i.e. discovered incidentally on a scan done for another reason) are thought to have no long-term consequences, patients with SPCs may suffer a number of chronic and acute symptoms, including headaches, vision and eye movement abnormalities, impairment of balance problems, speech and other cognitive problems, hydrocephalus, pineal apoplexy and even sudden death as a direct result of their pineal cyst (Nevins *et al.*, 2016)(Koziarski, Podgórski and Zieliński, 2019)(Májovský, Netuka and Beneš, 2017)(Fedorko, Zweckberger and Unterberg, 2018)(Kalani *et al.*, 2015)(Eide and Ringstad, 2017)(Choque-Velasquez *et al.*, 2019)(El Damaty *et al.*, 2019).

The exact incidence of SPCs is not known but, based on the numbers of contacts made with Pineal Cyst UK (PCUK) and the number of new patients we saw in the Eastern Region in 2019, we estimate that there are 200 new cases of SPC in the UK each year. The average age of patients with SPC is 35 years and the symptoms have major impact on patients' quality of life (QoL), including their role in family, work and society at large(Fedorko, Zweckberger and Unterberg, 2018).

Joanne Warren, an Australian SPC patient and a patient group moderator, has shared with us a survey of people with SPCs (79%) and pineal tumours (21%) the group conducted in 2018(Joanne Warren, 2018). The most frequently reported symptoms were headaches, visual symptoms, fatigue, sleep problems, hearing and balance problems, feeling of being disconnected from the surroundings, speech, memory and other cognitive problems. Very similar lists of symptoms have also been reported in the medical literature(Májovský, Netuka and Beneš, 2017)(Májovský, Netuka and Beneš, 2018)(Májovský, Netuka and Beneš, 2017)(Májovský, Netuka and Beneš, 2016)(Koziarski, Podgórski and Zieliński, 2019)(El Damaty *et al.*, 2019)(Kalani *et al.*, 2015)(Fedorko, Zweckberger and Unterberg, 2018)(Choque-

Velasquez *et al.*, 2019). These symptoms have a significant impact on patients' QoL and this has also been shown in the literature(Májovský, Netuka and Beneš, 2017)(Fedorko, Zweckberger and Unterberg, 2018). The most detrimental impact was recorded in "role functioning" domains, i.e. inability to fulfil roles in family, work and society at large. It has been reported that due to disability attributed to the SPCs, two thirds of patients were unable to carry out their normal activities and stay in work(Eide and Ringstad, 2017) (Fedorko, Zweckberger and Unterberg, 2018). Until recently, pineal cysts have rarely been considered to be the cause of patients' symptoms, unless causing hydrocephalus and/or Parinaud syndrome.

In the last five years eighth case series showing significant improvement in the symptoms and QoL after resection of SPCs have been published (Table 1). In these papers, improvement is reported in over 90% patients(Májovský, Netuka and Beneš, 2017)(Kalani *et al.*, 2015)(El Damaty *et al.*, 2019)(Koziarski, Podgórski and Zieliński, 2019)(Fedorko, Zweckberger and Unterberg, 2018)(Choque-Velasquez *et al.*, 2019)(Eide and Ringstad, 2017)(Choque-Velasquez *et al.*, 2019). Fedorko et al assessed QoL in a cohort of 12 patients with SPCs, which demonstrated a three-fold increase in the ability to carry out daily activities following surgery (Fedorko, Zweckberger and Unterberg, 2018). In another report, two thirds of patients who were unable to work prior, returned to work following surgery(Eide and Ringstad, 2017).

These studies showed that surgery can bring life-changing benefits to patients with SPC, signalling a potential paradigm shift in thinking about pineal cysts. However, given the relative novelty of these findings, the lack of understanding of the mechanism by which PC can cause symptoms as well as frequent co-existence with other medical conditions SPCs are frequently misdiagnosed and mistreated. In the cited survey by Warren(Joanne Warren, 2018) the majority of the responders had been assessed for and diagnosed with a variety of conditions, including migraine, cluster headaches, chronic fatigue syndrome, anxiety and depression. Not infrequently, when symptomatic management fails to bring improvement, the symptoms are labelled as "functional", i.e. having no material basis. This further compounds patients'

feelings of helplessness, isolation and dejection. Often, several layers of symptoms can accumulate, making the task of identifying the true root cause very difficult.

It is therefore essential to learn more about the safety and efficacy of surgery in controlling patients' symptoms and its effect on patients' quality of life, and to determine the predictive value of symptoms, radiological findings and their combination on patients quality of life after surgery. We propose a prospective observational cohort study that will prospectively collect clinical and radiological data on all patients with SPCs, whether they chose surgically or non-surgical (conservative) management. This will provide higher level of evidence regarding the safety and efficacy of improving patients' QoL than the published retrospective studies, but will also provide valuable information for predicting such improvement based on prospectively and systematically collected clinical and radiological data. Ultimately, a randomised control trial of surgery versus conservative management is likely to be required to provide definitive evidence for the efficacy of surgery and this prospective study will serve as a pilot for planning of such an RTC.

Year	Journal	1st author	Country	N	Symptom improvement*	Improvement in QoL
2015	J of Neurosurgery	Kalani	Australia	18	94%	NFI
2017	World Neurosurgery	Majovsky	Czech Republic	21	95%	NFI
2017	Acta Neurochirugica	Eide	Norway	15	100%	NFI
2019	Surgical Neurology International	Choque-Velasquez	Finland	60	100%	NFI
2019	Brit J Neurosurg	Koziarski	Poland	28	97%	NFI
2019	World Neurosurgery	El Dalmaty	Germany	43	95%	NFI
2019	J of Neurosurgery	Fedorko	Germany	12	100%	Yes
2019	Surg Neurol Internat	Choque-Velasquez	Finland	60	98%	NFI

Table 1. *Symptom improvement - % patients with symptoms improvement after surgery when compare to before surgery

NFI – not formally investigated, QoL – QoL

3. OBJECTIVES

3.1. PRIMARY OBJECTIVE

To assess the impact of surgery on quality of life, specifically patients' role functioning derived by the EORTC QLQ-C30 at one year after resection of symptomatic pineal cyst. Role functioning reflects activities in daily life, which is a higher-order outcome than symptoms, i.e. both symptoms and treatment have an impact on the level of functioning, a clear picture of the functioning of the patient. Role functioning was also the most relevant issue to patients seen in the clinic and the members of Pineal Cyst UK.

3.2. SECONDARY OBJECTIVES

3.2.1. Quality of Life – before and after surgery

We will determine the probability of improvement of overall QoL as well as each individual domain, as defined by the EORTC QLQ-C30 questionnaire (Appendix 1). This will be done by comparing pre-operative and post-operative assessments at 3, 12, 24 and 36 months following resection of SPC.

3.2.2. Symptoms – before and after surgery

We will determine the relative predictive values of individual symptoms and signs as well as their combinations in predicting improvement following resection of SPC. This will be done by comparing pre-operative and post-operative assessments at 3, 12, 24 and 36 months following resection of SPC.

3.2.3. Quality of life and symptoms of patients treated conservatively

We will assess Quality of Life and Symptoms of patient who chose conservative management at enrolment and at 3, 12, 24 and 36 months following the diagnosis of SPC. These data will also serve to compare baseline Quality of Life and Symptoms between patients managed surgically and conservatively. We will explore the differences of outcomes of patients who

choose conservative and surgical treatments with adjusting confounding factors. This cohort will also give us an idea about changes in severity of symptoms and QoL over time.

3.2.4. Cognitive status and emotional wellbeing – before and after surgery

As with most other resective brain surgery, SPC patients undergo detailed objective neuropsychological assessments before and after surgery. For the sake of the study this will be done systematically, with a standard protocol and in predefined intervals, i.e. before and 12 months after surgery. (Appendices 1-3)

3.3. EXPLORATORY SECONDARY OBJECTIVES

3.3.1. MRI-based anatomical and physiological predictors of outcome after surgery

The mechanisms underlying clinical presentation in patients with SPCs is largely unknown. This prospective cohort study offers a unique opportunity to collect data that will help our understanding of the physiological bases of patients' symptoms. We will use MR imaging to understand aetiology of symptoms of in patients with SPCs and to predict improvement of symptoms following surgery. In addition to searching for biomarkers, the data will be used in this exploratory part of the study. We will use MR imaging acquired for routine clinical reasons to determine anatomical (size, deformation of the tectum, relationship with great veins, relationship of size and tecto-callosal distance etc) and physiological (flow through the aqueduct of Sylvius, the deep cerebral venous system) predictors of improvement following resection of SPCs by comparing pre- and post-resection data.

3.3.2. Refinement of indications for surgical treatment of patients with SPCs

Patient clinical characteristics, symptoms and imaging data will be used in a model to predict symptom and QoL improvement following resection of SPCs.

4. PROJECT PLAN

We propose a single-centre, prospective controlled cohort study. Unlike previously published studies, which were retrospective cohort studies, this cohort will have *a priori* defined enrolment criteria, data points, outcomes and is powered to answer the study's questions. Given the patient numbers required (N=40) it is feasible to carry out the study in a single centre. The main advantages this design are maximum homogeneity of the study's variables and simplicity of its execution. However, if there is interest from other UK untints the study will be expanded under strict conditions of adherence to the study protocol and subject to governance approvals. This would accelerate recruitment.

As a result of Amendment 1, 21 consecutive patients who underwent an operation during the 5-year period prior to the commencement of the study (i.e. since 1st January 2016) and completed the required questionnaires and consented to participating in the study will be included in the total number of 40 surgically treated patients.

The primary outcome will be assessed once 40 surgically treated patients reach 12-months follow up. Since all the data required to assess the primary outcome was collected as a part of clinical care and will be available if patients consent for this to be used in the study, it may only be necessary to recruit 19 patients since the commencement of the study in order to achieve the primary objective as well as secondary objectives 3.2.1. and 3.2.2. of the study.

We will analyse and publish the result of the study with N=40. At that point, the cohort will consist of patients who underwent surgery before and after the commencement of the study. We will also evaluate the available data pertaining to the rest of the secondary of objectives (3.2.3, 3.2.4) and exploratory objectives (3.3.1 and 3.3.2). These data will be less complete and results less informative, but we believe this is a practically and ethically beneficial compromise to mitigate the negative impact of the COVID-19 pandemic.

4.1. INCLUSION CRITERIA

- Age =/> 16 years

- Pineal cyst > 9mm in maximum diameter (Májovský et al., 2017)

- Symptom category B (total score ≥5) according to Eide and Ringstad(Eide and Ringstad, 2017) – Figure 1_Eide symptoms

- Willingness and ability to comply with study specific procedures and completion of QoL and symptoms questionnaires.

- Written informed consent

Acta Neurochir (2017) 159:349–361

Table 1 A scale for grading of symptoms in patients with pineal cysts

Symptom	Severity	Score
Headache	No	0
	Minor-moderate	1
	Severe	2
Nausea/vomiting	No	0
	Yes	1
Dizziness	No	0
	Yes	1
Visual disturbances	No	0
	Yes	1
Episodic loss of consciousness	No	0
	Yes	1
Lethargy/fatigue	None-moderate	0
	Severe	1
Cognitive impairment	No	0
	Minor-moderate	1
	Severe	2
Transient neurological deficits	No	0
	Yes	1
Total score		0/10 – 10/10

Symptom category A (none-moderate), 0–4/10 scores; symptom category B (much-severe), 5–10/10 scores

4.2. EXCLUSION CRITERIA

- Evidence for alternative diagnoses/pathological processes causing presenting symptoms

4.3. RECRUITMENT STRATEGY AND SCREENING

When patient receive a diagnosis of a symptomatic pineal cyst (SPC) during a routine neurosurgical clinic consultation at the Addenbrooke's Hospital in Cambridge they will be informed about the possibility of taking part in this study by the patient clinical care team.

Patients referred to neurosurgical clinic for evaluation of their pineal cyst, either symptomatic or asymptomatic will be seen as per current practice. If a diagnosis of a symptomatic pineal cyst (SPC) is made (SPC > 9mm, Eide and Ringstad symptom B category), patients will be screened against inclusion and exclusion criteria (see Section 4.1 and 4.2). If fulfilling the entry criteria, patients will be offered participation in the study. Patients will be advised about the aims, expected benefits and risks of surgery as per current practice. Patients who request surgery will undergo an operation as per existing care pathway. Patients who chose not to undergo surgery will pursue conservative management as per existing care pathway. Interested patients regardless the pathway they have chosen will be explained the study and will be given a Participant Information Sheet (PIS). If they wish to join the study they will sign a Consent Form for participation in the study.

We anticipate that recruitment of the required number of patients treated by surgery will take approximately two years (N=40). The follow up for the primary outcome measure will take additional year. The study specific follow up assessments will be performed at one year after surgery for the primary efficacy outcome measure and SPC-related symptoms review. Given the relatively low burden posed by administering and filling in of the of the questionnaires, one early (3 months following surgery) and two late (at 2 and 3 years after surgery) surveys of symptom control and QoL will be performed (see also attached Flow Chart – Appendix 7 and Flow Table – Appendix 8).

Eligible participants who chose not to undergo surgery (this is an entirely clinical and not a research-related decision) will be recruited the non-surgery (conservative treatment) cohort (see also attached Flow Chart – Appendix 7 and Flow Table – Appendix 8). This is to

investigate/explore the QoL difference between surgery and non-surgery patients, both at diagnosis (baseline) and 3, 12, 24 and 36 months after treatment (one of the secondary objectives).

4. 4. NUMBERS AND METHODS OF ANALYSIS.

4.4.1. Primary outcome and sample size

The primary efficacy outcome measure is the change of role functioning scale of the EORTC QLQ c30 questionnaire at 12 months after surgery.

It is anticipated that a considerable clinical effect will be achieved, that is, an improvement of at least 20 points in a 0-100 scale in the role functioning scale. Using the estimated standard deviation of 42 at pre-surgery (retrospectively assessed) and 18 at 6 months post-surgery (prospectively assessed)(Fedorko, Zweckberger and Unterberg, 2018), with a conservative assumption of the correlation coefficient between pre- and post-surgery scores being 0.5, the estimated standard deviation of the difference between pre and post-surgery is 36. With a 5% significance level, 90% power, 37 participants are required to detect the 20 points using a paired t-test.

Allowing for some non-compliance and to maximise the yield from the secondary outcome measures we aim to recruit 40 participants. The assumptions used for sample size estimation will be monitored by the Study Steering Committee (see below). The conservatively treated patients, i.e. those who chose not to undergo surgery, will be recruited until the completion of surgically-treated patients. No power calculations are required as outcome measures related to the conservatively treated (non-surgery) cohort are among secondary objectives of this study.

4.4.2. Secondary outcomes

The secondary outcomes are:

1. Change in all in items of QoL as defined by the EORTC QLQ-C30 questionnaire before and at 3, 12, 24 and 36 months following resection of SPC

2. Change of individual symptoms before and at 3, 12, 24 and 36 months following resection of SPC

3. Change in the results neuropsychological tests at 12 months following surgery when compared to baseline (before surgery)

4. Difference in severity of symptoms and QoL at baseline between patients who chose surgical and non-surgical treatment

5. Assessment of symptoms and QoL over (at diagnosis and 3, 12, 24 and 36 months later) in patients who chose not to undergo surgery

6. Development of a model to identify anatomical (based in MRI scans) and clinical (symptoms) predictors of improvement of QoL after surgery for SPC

4.4.3. Exploratory outcomes

- development of a model to identify anatomical (MRI) and clinical (symptoms) predictors of improvement of QoL after surgery for SPC

4.4.4. Statistical analyses

The paired t-test will be applied to the primary efficacy analysis for all patients received surgery treatment. A detailed statistical analysis plan will be drafted prior to any analyses performed.

4.5. STUDY PROCEDURES

Individual patient participation – overview (see also Flow Chart and Flow Table):

4.5.1. Screening

a. Patients referred to neurosurgical clinic that are diagnosed with symptomatic pineal cyst (SPC) will be screened against inclusion and exclusion criteria. The treatment will be offered and carried out as per current evidence-based clinical practise. Whether patients chose surgery or conservative management, they will be eligible to be enrolled in the study.

b. Information about the study will be provided to eligible patients by a member of the research team after the end of the first neurosurgery clinic appointment. This can occur face-to-face or over the telephone.

c. Informed consent to participate in the study will be obtained by a member of the research team after the patients have considered the relevant information about participation. Online completion of consent forms will be considered, in keeping with the reduction of face-to-face appointments due to the pandemic. Paper consent forms will also be available.

As a result of Amendment 1, patients who had undergone resection of the pineal cyst prior to commencement of the study and have completed symptoms and quality of life questionnaires will be contacted via telephone by a member of the research team. All patients are currently under follow up. The study and their potential role as participants in the study will be explained and if patients express their interest in participating in the study, Patient Information Sheet and Consent Form will be send to them. They will be offered an appointment to discuss this in person or, if they prefer, they can sign the consent form in the presence of a witness and mail this back to the research team.

4.5.2. Symptoms and QoL questionnaires (see Appendices 1-4)

a. Patients who chose surgical treatment

Study participants will undergo surgery as per normal clinical pathway. The participants will be asked to fill in symptom and quality of life (QoL) questionnaires at the entry to the study

(at diagnosis) and at 3, 12, 26 and 36 months after surgery. The participants will be given the options of filling the questionnaire on the paper or on-line.

b. Patients who chose conservative management

The participants will be asked to fill in symptom and quality of life (QoL) questionnaires at the entry to the study (at diagnosis) and at 3, 12, 26 and 36 months after the diagnosis. The participants will be given the options of filling the questionnaire on the paper or on-line.

Once enrolled patients will participate in the study for 3 years, although each participant will be able to leave the study at any time without giving a reason. This will in no way affect their clinical management. This includes the option for patients to choose to undergo surgery at any time even if they have initially elected to pursue conservative management.

4.6. INFORMED CONSENT

Informed consent will be taken from patients in a neurosurgical clinic after the diagnosis of a symptomatic pineal cyst is made, patient expresses interest in taking part in the study and passes through the inclusion and exclusion criteria as eligible for participation in the study.

A member of the research team will take informed consent. The person taking informed consent will be experienced in assessing capacity and aware of the ethical issues involved.

Participants will be provided with the current version of the REC-approved Patient Information Sheet for review.

Participants will be given the opportunity to ask any questions and only once all of their queries have been answered will the consent be taken by the Principal Investigator or suitably qualified, delegated member of the clinical care team. Patients will be informed that they can withdraw their consent at any time without giving reasons for doing so.

The process of obtaining a consent from patients who underwent an operation prior to the commencement of the study (see Amendment 1) is described in section 4.5.1. The Consent Form itself will be identical to those used for patients recruited after the date of commencement of the study. A tailored Patient Information Sheet will provide these patients with relevant information about study participation.

4.7. STUDY ASSESSMENTS

Assessment methods:

4.7.1. Questionnaires. – see above

4.8. DEFINITION OF END OF STUDY

The study will begin with at a given start date and will conclude after the last patient completes three-year questionnaire. (see also Flow Chart and Flow Table in the Appendix 7 and 8, respectively):

5. INTERVENTIONS

The only interventions in this study are questionnaires that the patients will be asked to fill in. Please see section 4.6.

As a result of Amendment 1, the patients who had undergone surgery prior to the commencement of the study have already filled in some or all required questionnaires for the purpose of clinical management and will only be asked for a permission to use them for the purpose of this research study. In addition, they will be prospectively asked to fill in questionnaires that are due at the predefined future time points.

6. SAFETY REPORTING

6.1 Safety data collection

This is an observational study where the only interventions are questionnaires and collection of clinical data. Nevertheless, this study presents an opportunity to systematically monitor surgical complications whose governance will fall into the realm of clinical rather than research governance. As such, we don't anticipate research-related events. Nevertheless, we will have system in place whereby any relevant events will feed into existing departmental governance structure.

7. STUDY COMMITTEES

Study Management Group

The Chief Investigator (CI) together with the research team are responsible for the day-to-day running of the study as detailed in study-specific procedures. They will work together as the Study Management Group and will meet monthly.

Study Steering Committee

It will consist of one independent neurosurgeon as the chair, one patient representative and one member of Study Management Group. The Study Steering Committee will monitor the conduct and progress of the study, including recruitment, data completeness and deviations from the protocol. The Study Steering Committee will meet every 6 months while participants are on treatment.

1. ETHICAL & REGULATORY CONSIDERATIONS

Consent

The Informed Consent form must be approved by the REC and must be in compliance with GCP, local regulatory requirements and legal requirements. The investigator must ensure that each study participant is fully informed about the nature and objectives of the study and possible risks associated with their participation.

The investigator will obtain written informed consent from each patient before any study-specific activity is performed. The informed consent form used for this study and any change made during the course of this study, must be prospectively approved by the REC. The investigator will retain the original of each patient's signed informed consent form.

Should a patient require a verbal translation of the study documentation by a locally approved interpreter/translator, it is the responsibility of the individual investigator to use locally approved translators.

Ethical committee review

Before the start of the study or implementation of any amendment we will obtain approval of the study protocol, protocol amendments, informed consent forms and other relevant documents (e.g. advertisements and GP information letters, if applicable) from the REC. All correspondence with the REC will be retained in the Study Master File/Investigator Site File.

Annual reports will be submitted to the REC in accordance with national requirements. It is the Chief Investigator's responsibility to produce the annual reports as required.

Study protocol amendments

Protocol amendments must be reviewed and agreement received from the Sponsor for all proposed amendments prior to submission to the REC.

The only circumstance in which an amendment may be initiated prior to REC approval is where the change is necessary to eliminate apparent, immediate risks to the patients (Urgent Safety Measures). In this case, accrual of new patients will be halted until the REC approval has been obtained.

Declaration of Helsinki and Good Clinical Practice

The study will be performed in accordance with the spirit and the letter of the declaration of Helsinki, the conditions and principles of Good Clinical Practice, the protocol and applicable local regulatory requirements and laws.

GCP training

All study staff must hold evidence of appropriate GCP training or undergo GCP training prior to undertaking any responsibilities on this study. This training should be updated every 2 years or in accordance with your Trust's policy.

7. ETHICS

7.1. Participant Confidentiality

The study staff will ensure that the participants' anonymity is maintained. Each participant will be allocated a unique study number and will be identified only by this number. The information linking each number and the details of each participant will be stored securely in a separate file. All data will be identified by the participant number only. All documents will be stored securely and only accessible by study staff and authorised personnel. The study will comply with the Data Protection Act which requires data to be anonymised as soon as it is practical to do so.

7.2. Other Ethical Considerations

Recruitment will be performed by a consultant neurosurgeon experienced in performing pineal region surgery. There will be no therapeutic promises, coercion or inducement of any sort.
A minimum of personal data and no participant-identifiable data will be gathered.
The only interventions are questionnaires and most questions are expansions of existing non-standardised symptom and lifestyle clinical interview caried in routine clinical practise.

8. DATA HANDLING AND RECORD KEEPING

All patient data will be anonymised. The participants will be identified by a study specific participant number. The information linking each number and the details of each participant will be stored securely in a separate file. All data will be identified by the participant number only. All data will be stored securely on Cambridge University Hospitals (CUH) NHS Trust computing clusters only. This is secure access and backed up automatically. No data will be stored on personal computers.

9. SPONSORSHIP, FINANCING AND INSURANCE

The study is jointly sponsored by the Cambridge University Hospitals NHS Foundation Trust.

All procedures will be done in NHS premises as part of the routine clinical care for patients.

Financing: No funding is required for this study as the amount of 'research-related' work is minimal and this work will be done on volunteer bases by the study participants and investigators.

10. POTENTIAL EXPANSIONS OF THE STUDY

This study may be expanded to a small number (one or two) of additional UK-based neurosurgical centres. Amendments to protocols and ethics application will be submitted as required prior to conducting any such potential expansions.

11. THE TEAM

The Team of experienced academic clinicians, scientists and a patient representative has been assembled to develop and execute a study.

Investigators

Miss Anna Hill, Patient Representative, Pineal Cyst UK

Prof Peter Hutchinson, BSc MBBS PhD FRCS(SN) – Consultant Neurosurgeon, University of Cambridge, Cambridge University Hospitals NHS Trust

Mr Alexis Joannides, MA PhD MB BChir FRCS(SN) – Consultant Neurosurgeon, University of Cambridge, Cambridge University Hospitals NHS Trust

Mr Angelos Kolias, MD MSc PhD FRCS(SN) – Clinical Lecturer, University of Cambridge, Cambridge University Hospitals NHS Trust

Dr Riccardo Masina, MB.BChir – Honorary Foundation Officer, University of Cambridge, Cambridge University Hospitals NHS Trust

Dr Wendi Qian, PhD – Senior Statistician, Cambridge Clinical Trials Unit, University of Cambridge

Dr Amber Steele, PhD – Research Advisor, Cambridge Research Design Services, National Institute for Health Research

Collaborators

Dr Timothy Ham, MB BS BSc PhD – Consultant Neurologist, Cambridge University Hospitals

Prof Manohar Bance – Professor of Otolaryngology, Consultant ENT Surgeon, University of Cambridge. Internationally recognised expert and leading researcher in the field of hearing and balance. Hearing and balance are among the important symptoms.

Many patients present with vertigo and hearing problems and these patients are often referred to ENT specialists. To assure highest quality of relevant clinical data, rigorous and

systematic evaluation of each patient by an ENT surgeon with interest in hearing and balance is vital.

Prof Marek Czosnyka, DSc, PhD - Brain Physics, University of Cambridge. Internationally recognised leader in the field of brain physics, especially in cerebrospinal fluid (CSF) dynamics and intracranial pressure (ICP) monitoring and analysis. Discovered and described a number of fundamental principles of CSF and ICP physiology, their measurement and use in the clinical practise. MC's team expertise will be useful in trying to understand the pathogenesis of symptoms in SPCs.

Dr Linda Dirven, PhD – Senior Researcher, Leiden University Medical Center, The Netherlands. Linda Dirven is an internationally recognised leader in the measurement of outcome in neurological and neurosurgical conditions. Developed and validated tools to measure symptoms and quality of life (QoL). LD advised on selection of the most relevant tools to measure changes in quality of life and symptoms following surgery in this patient population.

Dr Tomasz Matys, MD PhD, academic neuro-radiologist with special interest in MR imaging of brain tumours, neuro-degenerative conditions and cerebrospinal fluid.

TM's expertise has been used in the selection of relevant imaging sequences and TM will lead analysis and interpretation of imaging data.

Miss Brinda Muthusamy, MBChB, MRCP UK, FRCOphth Neurosciences, Ophthalmology Consultant Neuro-ophthalmologist with extensive clinical experience in conditions related to neurosurgical practise, including brainstem and pineal region pathology. Has evaluated close to 100 patients with symptomatic pineal cysts (most referred by the Lead Investigator). Visual symptoms are the second most common symptoms in patients with pineal cysts and neuroophthalmological expertise is vital to detect and record this appropriately.

Neuropsychology team led by Dr Emma Woodberry with participation by Dr Alexa McDonald, Dr Priya Varma. The neuropsychology team is recognised by the CQC as providing an 'outstanding' service to patients. The team works closely with the brain tumour

team, routinely evaluating patients before and after surgery. The team has the expertise and structure to create a comprehensive and objective snapshot of the neuropsychological status of patients including their cognitive functioning and

emotional wellbeing. As patients with SPCs frequently develop neuropsychological symptoms, it is vital to record these and compare the pre-operative and postoperative status.

13. REFERENCES

Choque-Velasquez, J. *et al.* (2019) 'The microsurgical management of benign pineal cysts: Helsinki experience in 60 cases', *Surgical Neurology International*, 10, p. 103. doi: 10.25259/SNI-180-2019.

Eide, P. K. and Ringstad, G. (2017) 'Results of surgery in symptomatic non-hydrocephalic pineal cysts: role of magnetic resonance imaging biomarkers indicative of central venous hypertension.', *Acta neurochirurgica*, 159(2), pp. 349–361. doi: 10.1007/s00701-016-3029-4.

El Damaty, A. *et al.* (2019) 'Pineal Cyst without Hydrocephalus: Clinical Presentation and Postoperative Clinical Course After Infratentorial Supracerebellar Resection', *World Neurosurgery*, 129, pp. e530–e537. doi: 10.1016/j.wneu.2019.05.200.

Fedorko, S., Zweckberger, K. and Unterberg, A. W. (2018) 'Quality of life following surgical treatment of lesions within the pineal region', *Journal of Neurosurgery*, pp. 1–10. doi: 10.3171/2017.7.JNS17260.

Golzarian, J. *et al.* (1993) 'Pineal cyst: normal or pathological?', *Neuroradiology*, 35(4), pp. 251–253. doi: 10.1007/bf00602604.

Joanne Warren (2018) 'Pineal Cysts and Tumours Independent Survey of Symptoms 2018'.

Kalani, M. Y. S. *et al.* (2015) 'Pineal cyst resection in the absence of ventriculomegaly or Parinaud's syndrome: clinical outcomes and implications for patient selection', *Journal of Neurosurgery*, 123(2), pp. 352–356. doi: 10.3171/2014.9.JNS141081.

Koziarski, A., Podgórski, A. and Zieliński, G. M. (2019) 'Surgical treatment of pineal cysts in non-hydrocephalic and neurologically intact patients: selection of surgical candidates and clinical outcome', *British Journal of Neurosurgery*, 33(1), pp. 37–42. doi: 10.1080/02688697.2018.1530731.

Májovský, M., Netuka, D. and Beneš, V. (2016) 'Clinical management of pineal cysts: a worldwide online survey.', *Acta neurochirurgica*, 158(4), pp. 663–669. doi: 10.1007/s00701-016-2726-3.

Májovský, M., Netuka, D. and Beneš, V. (2017) 'Conservative and surgical treatment of patients with pineal cysts: A prospective case series of 110 patients.', *World neurosurgery*, 105, pp. 199–205. doi: 10.1016/j.wneu.2017.05.155.

Májovský, M., Netuka, D. and Beneš, V. (2018) 'Is surgery for pineal cysts safe and effective? Short review', *Neurosurgical Review*, 41(1), pp. 119–124. doi: 10.1007/s10143-017-0876-2.

Mamourian, A. C. and Towfighi, J. (1986) 'Pineal cysts: MR imaging', *AJNR. American journal of neuroradiology*, 7(6), pp. 1081–1086.

Nevins, E. J. *et al*. (2016) 'Incidental Pineal Cysts: Is Surveillance Necessary?', *World neurosurgery*, 90, pp. 96–102. doi: 10.1016/j.wneu.2016.02.092.

3. Patient information sheet

Department of Clincial Neurosciences
Box 166
Addenbrooke's Hospital
Cambridge
CB2 0QQ

INFORMATION SHEET:
PATIENTS

Version number: 1.2
Date: 28 June 2021

Title of Project: **Assessment of changes in symptoms and quality of life after surgical treatment of patients with symptomatic pineal cyst - a prospective observational cohort study**

You are being invited to take part in a research study. Before you decide it is important for you to understand why the research is being done and what it will involve. Please take time to read the following information carefully and discuss it with others if you wish.

Thank you for reading this.

Part 1 *tells you the purpose of this study and what will happen to you if you take part.*
Part 2 *gives you more detailed information about the conduct of the study.*

Please ask if anything is not clear or if you would like more information. Take time to decide whether or not to take part.

Part 1

What is the purpose of this study?
Following our discussion in the clinic you will know that pineal cysts (PCs) are common and that only some PCs are thought to cause symptoms. Until only quite recently, it had been the understanding of the medical community that PCs don't cause symptoms. However, several studies published since 2015 have shown that the majority of patients with symptoms (headaches; visual disturbances; balance and hearing problems; memory, speech and other cognitive impairment etc) improve following surgical removal of the cyst. These are early studies, based on review of clinical records of relatively small number of patients. As helpful as these

studies are, a higher level of clinical evidence is required to reduce the uncertainty about the role of surgery in the management of symptoms of patients with symptomatic pineal cysts (SPCs). The aim of our study is to collect comprehensive and beforehand agreed information about symptoms and quality of life of patients with SPCs. Some patients will choose to undergo surgery while others will choose to be treated by non-surgical means. Comparing the information about symptoms and quality of life from before and after surgery will not only help our understanding of the value of surgery in SPCs, but will also help calculating the probability of each symptom improving following surgery. Questionnaires from patients who choose not to undergo surgery will help improve our understanding of symptoms of patients with SPCs over time and also see whether there is a difference in the type and severity symptoms between patients who choose to undergo surgery and those who prefer non-surgical management.

Why have I been invited to participate in the study?
We are inviting all adult patients with the diagnosis of symptomatic pineal cysts (SPCs).

Do I have to take part?
It is up to you to decide. We will describe the study and go through this Information Sheet, which we will then give to you. If you feel you would like to participate in this study, we will then ask you to sign a consent form to show that you have agreed to take part. You are free to withdraw your consent at any time, without giving a reason. Not taking part in of withdrawing from the study will not affect in any way they type of treatment or the standard of care you will receive.

What will happen to me if I take part?
Your preparation for surgery, surgery and aftercare will essentially be the same as if you did not participate in the study. The only activity that relates to the study are Quality of Life and Symptom questionnaires that we will ask you to fill in before surgery (or at diagnosis, if you chose not to undergo surgery) and at 3, 12, 24 and 36 months following surgery (following diagnosis, if you chose not to undergo surgery). These questionnaires will also help us with following your progress in more detail.

Will taking part interfere with my treatment?
Taking part will have no effect on the treatment you will receive. Likewise if you decide not to proceed with this study or leave the study at any point, it will not alter the treatment that you will receive.

What are the possible disadvantages and risks of taking part?
There are no real disadvantages in taking part in the study as it doesn not influence the course of your treatment.

Will my GP be informed?
We won’t routinely inform your GP unless you specifically ask us to. We believe that there is no benefit in routinely informing GPs because participation in the study will have no immediate or long-term effects on your health.

What are the possible benefits of taking part?
There will be no direct benefit to patients as a result of participation in the study. However, we hope the detailed information about your symptoms and quality of life will help us better understand your symptoms and related quality of life as well as patients with symptomatic pineal cysts in general.

What if there is a problem?
Any complaint about the way you have been dealt with during the study or any suggestion of possible harm will be investigated and addressed as required. The detailed information on this is given in Part 2.

Will my taking part in the study be kept confidential?
Yes. We will not inform anyone of your participation in the study without your consent. All information gathered about you during the study will be kept confidential. The details are included in Part 2.

Contacts for further information.

Mr. Thomas Santarius, MD PhD FRCS(SN)
Consultant Neurosurgeon,
Department of Neurosurgery,
Division of Clinical Neurosciences,
Addenbrooke's Hospital,
Cambridge CB2 0QQ.

Tel.: 01223-586858
E-mail: maria.harrington@addenbrookes.nhs.uk

For further independent assistance, please contact:

Patient **A**dvice & **L**iaison **S**ervice (PALS)
Box 53,
Cambridge University Hospitals NHS Foundation Trust,
Hills Road,
Cambridge CB2 0QQ

Tel: 01223 216 756
From bedside Patientline: *801

E-mail: pals@addenbrookes.nhs.uk

This completes part 1 of the Information sheet.

Part 2

What if relevant new information becomes available?
Sometimes we get new information about the problem being studied. If this happens, your research doctor will tell you and discuss whether you should continue in the study. If you decide not to carry on, your research doctor will make arrangements for your care to continue. If you decide to continue in the study they may ask you to sign an updated consent form. If the study is stopped for any other reason, we will tell you. This will not affect your care in any way.
This is, however, an extremely unlikely consideration in this study.

What will happen if I don't want to carry on with the study?
You can withdraw from the study at any time without giving a reason. Information that was already collected may still be used, unless you will ask not to use it. Your withdrawal will not affect your care in any way.

What if I am unhappy with things or something goes wrong?
If you have concerns about any aspect of this study, you should ask to speak to the researchers who will do their best to answer your questions (please see contact details at the end of Part 1). If you remain unhappy and wish to complain formally, you can do this through the NHS Complaints Procedure. Details can be obtained from the Addenbrooke's Patient Advice and Liaison Service (PALS).

Are there compensation arrangements if something goes wrong?
In the unlikely event of anything untoward happening as a result of you taking part in the study, all patients registered with Cambridge University Hospitals NHS Foundation Trust are covered by the Trust's indemnity. In addition, clinical staff carry their own personal insurance. In the event that something does go wrong and you are harmed during the research and this is due to someone's negligence then you may have grounds for a legal action for compensation against Cambridge University Hospitals NHS Foundation Trust, but you may have to pay your legal costs. The normal National Health Service complaints mechanisms will still be available to you (if appropriate).

Who is organising and funding the research?
The study is organised by a research group from the Departments of Neurosurgery, National Institute for Health Research and the Cambridge Clinical Trials Unit. The study is run by highly experienced medical scientists who do all the work related to this study on voluntary (unpaid) basis.

Who has looked at and approved the study?
All research in the NHS is looked at by an independent group of people called the Research Ethics Committee. The Committee is setup to review each project carefully to protect your safety, rights, wellbeing and dignity. This study has been reviewed and given favourable opinion by the Research Ethics Committee.

Confidentiality – who will have access to the data?
All information which is collected about you during the course of the research will be kept strictly confidential and any clinical data will be fully anonymised before being used for research. At present we have no plans to share any of these anonymised data with data with researchers at other institutions. However, we may potentially do this in future if we were to set up a larger, multi-institutional study to better understand your condition and its treatment. If we were to do this, i.e. contribute your fully anonymised data to national or international studies, we would first seek approval of the Ethics Committee.

MRI scans that are taken as part of your routine clinical care are saved with personal identifiable information such as your name. However, this will be used for research purposes only after being anonymised and will be unidentifiable.

Data collected during the study will be stored on a secure network belonging to the Cambridge University Hospitals (CUH). All data will by anonymised and only members of the research group at the Departments of Radiology and Neurosurgery will have access to the data. Cambridge University Hospitals (CUH) is deemed to be the Data Controller and all enquiries concerning access to the data should be addressed to it. The Administrator of the Centre will be able to tell you the name and address of the relevant officer.

What will happen to the study results?
The data will be kept securely for a minimum of 10 years and possibly indefinitely in the Departments of Neurosurgery in accordance with good research practice. It is our aim to share the results of the study with other scientists and health care professionals. The results will therefore be published in peer-reviewed scientific journals, internal reports, conference presentation, publication on websites and other forms of scientific dissemination. All disseminated results will be anonymised and unidentifiable.

Will video/audio tapes be used?
No.

You may withdraw from the study at any time without explaining why and it will not affect the present or future treatment in any way.

GCPR statement
Cambridge University Hospitals NHS Foundation Trust (CUHNFT) is the Sponsor for this study based in the United Kingdom. They will be using information from [you and/or your medical records] in order to undertake this study and will act as the data controller. This means that this organisation is responsible for looking after your information and using it properly. CUHNFT will keep identifiable information about you for x years after the study has finished/ until x].Your rights to access, change or move your information are limited, as we need to manage your information in specific ways in order for the research to be reliable and accurate. If you withdraw from the study, we will keep the information about you that we have already obtained. To safeguard your rights, we will use the minimum personally-identifiable information possible. You can find out more about how we use your information using the following links:

For Cambridge University Hospitals NHS Foundation Trust, please visit: https://www.cuh.nhs.uk/corporate-information/about-us/our-responsibilities/looking-after-your-information, or email the Data Protection Officer at: gdpr.enquiries@addenbrookes.nhs.uk

This research study has been approved by the Research Ethics Committee.

Contacts for further information

Mr. Thomas Santarius, MD PhD FRCS(SN)
Consultant Neurosurgeon,
Department of Neurosurgery,
Division of Clinical Neurosciences,
Addenbrooke's Hospital,
Cambridge CB2 0QQ.

Tel.: 01223-586858
E-mail: maria.harrington@addenbrookes.nhs.uk

For further independent assistance, please contact:

Patient **A**dvice & **L**iaison **S**ervice (PALS)
Box 53,
Cambridge University Hospitals NHS Foundation Trust,
Hills Road,
Cambridge CB2 0QQ

Tel: 01223 216 756
From bedside Patientline: *801

E-mail: pals@addenbrookes.nhs.uk

Thank you for considering taking part in this study. Our research depends entirely on the goodwill of potential volunteers such as you. If you require any further information, we will be pleased to help you in any way we can.

4. Literature review and meta-analysis

Systematic review and meta-analysis of existing literature:

We have conducted a systematic review and meta-analysis of the existing literature on surgical management of non-hydrocephalic symptomatic pineal cysts. For a detailed report of results and methodological approach, please refer to our publication on *Acta Neurochirurgica* (Masina et al, 2022).

Briefly, the search strategy combined relevant medical subject headings (MeSH) and keywords. The search strategy has been drafted by RM and reviewed by TS – it is available below.

Reports had to be searchable through the PubMed and SCOPUS databases via the search terms "Pineal Cyst" and "Surgery" as of March 2021. No constraints on study design, year of publication or publication status were imposed. From the 1537 unique records identified by this search, screening for relevance by title and abstract resulted in 1468 articles being excluded. Of the remaining 69 articles selected for full-text evaluation, a total of 46 were excluded (Figure_1 of Masina et al 2022) for the following reasons: pineal lesion not a pineal cyst (13); the study not allowing to extract the cases without hydrocephalus from the reported cohort (11); radiologically confirmed hydrocephalus (10); the study being a review article (6); surgical outcome not reported (3); report of surgical technique only (1); no surgical intervention (1); the report being an editorial letter.

The authors of all case series with N>5 published since 2000 were successfully contacted for the latest version of their data. As a result, the authors from 3 studies that were excluded because of missing data were able to provide the necessary information, which resulted in their work meeting all inclusion and exclusion criteria.

Search strategy:

(A) Pubmed:

Query: (pineal cyst) AND (surgery) Filters: Humans

(("pineal gland"[MeSH Terms] OR ("pineal"[All Fields] AND "gland"[All Fields]) OR "pineal gland"[All Fields] OR "pineal"[All Fields] OR "pineale"[All Fields] OR "pineals"[All Fields]) AND ("cysts"[MeSH Terms] OR "cysts"[All Fields] OR "cyst"[All Fields] OR "neurofibroma"[MeSH Terms] OR "neurofibroma"[All Fields] OR "neurofibromas"[All Fields] OR "tumor s"[All Fields] OR "tumoral"[All Fields] OR "tumorous"[All Fields] OR "tumour"[All Fields] OR "neoplasms"[MeSH Terms] OR "neoplasms"[All Fields] OR "tumor"[All Fields] OR "tumour s"[All Fields] OR "tumoural"[All Fields] OR "tumourous"[All Fields] OR "tumours"[All Fields] OR "tumors"[All Fields]) AND ("surgery"[MeSH Subheading] OR "surgery"[All Fields] OR "surgical procedures, operative"[MeSH Terms] OR ("surgical"[All Fields] AND "procedures"[All Fields] AND "operative"[All Fields]) OR "operative surgical procedures"[All Fields] OR "general surgery"[MeSH Terms] OR ("general"[All Fields] AND "surgery"[All Fields]) OR "general surgery"[All Fields] OR "surgery s"[All Fields] OR "surgerys"[All Fields] OR "surgeries"[All Fields])) AND (humans[Filter])

Translations

pineal: "pineal gland"[MeSH Terms] OR ("pineal"[All Fields] AND "gland"[All Fields]) OR "pineal gland"[All Fields] OR "pineal"[All Fields] OR "pineale"[All Fields] OR "pineals"[All Fields]

cyst: "cysts"[MeSH Terms] OR "cysts"[All Fields] OR "cyst"[All Fields] OR "neurofibroma"[MeSH Terms] OR "neurofibroma"[All Fields] OR "neurofibromas"[All Fields] OR "tumor's"[All Fields] OR "tumoral"[All Fields] OR "tumorous"[All Fields] OR "tumour"[All Fields] OR "neoplasms"[MeSH Terms] OR "neoplasms"[All Fields] OR "tumor"[All Fields] OR "tumour's"[All Fields] OR "tumoural"[All Fields] OR "tumourous"[All Fields] OR "tumours"[All Fields] OR "tumors"[All Fields]

surgery: "surgery"[Subheading] OR "surgery"[All Fields] OR "surgical procedures, operative"[MeSH Terms] OR ("surgical"[All Fields] AND "procedures"[All Fields] AND "operative"[All Fields]) OR "operative surgical procedures"[All Fields] OR "general surgery"[MeSH Terms] OR ("general"[All Fields] AND "surgery"[All Fields]) OR "general surgery"[All Fields] OR "surgery's"[All Fields] OR "surgerys"[All Fields] OR "surgeries"[All Fields]

Number of results: 1533

Date searched: 31/03/2021

(B) SCOPUS:

Query: (TITLE-ABS-KEY(pineal cyst))

Number of results: 735

Date searched: 31/03/2021

5. EORTC-QLQ-C30 Questionnaires

EORTC QLQ-C30 (version 3)

We are interested in some things about you and your health. Please answer all of the questions yourself by circling the number that best applies to you. There are no "right" or "wrong" answers. The information that you provide will remain strictly confidential.

Please fill in your initials: |_|_|_|_|
Your birthdate (Day, Month, Year): |_|_|_|_|_|_|_|_|
Today's date (Day, Month, Year): 31 |_|_|_|_|_|_|_|_|

	Not at All	A Little	Quite a Bit	Very Much
1. Do you have any trouble doing strenuous activities, like carrying a heavy shopping bag or a suitcase?	1	2	3	4
2. Do you have any trouble taking a long walk?	1	2	3	4
3. Do you have any trouble taking a short walk outside of the house?	1	2	3	4
4. Do you need to stay in bed or a chair during the day?	1	2	3	4
5. Do you need help with eating, dressing, washing yourself or using the toilet?	1	2	3	4

During the past week:

	Not at All	A Little	Quite a Bit	Very Much
6. Were you limited in doing either your work or other daily activities?	1	2	3	4
7. Were you limited in pursuing your hobbies or other leisure time activities?	1	2	3	4
8. Were you short of breath?	1	2	3	4
9. Have you had pain?	1	2	3	4
10. Did you need to rest?	1	2	3	4
11. Have you had trouble sleeping?	1	2	3	4
12. Have you felt weak?	1	2	3	4
13. Have you lacked appetite?	1	2	3	4
14. Have you felt nauseated?	1	2	3	4
15. Have you vomited?	1	2	3	4
16. Have you been constipated?	1	2	3	4

SPECIMEN

Please go on to the next page

**During the past week:**	**Not at All**	**A Little**	**Quite a Bit**	**Very Much**
17. Have you had diarrhea?	1	2	3	4
18. Were you tired?	1	2	3	4
19. Did pain interfere with your daily activities?	1	2	3	4
20. Have you had difficulty in concentrating on things, like reading a newspaper or watching television?	1	2	3	4
21. Did you feel tense?	1	2	3	4
22. Did you worry?	1	2	3	4
23. Did you feel irritable?	1	2	3	4
24. Did you feel depressed?	1	2	3	4
25. Have you had difficulty remembering things?	1	2	3	4
26. Has your physical condition or medical treatment interfered with your family life?	1	2	3	4
27. Has your physical condition or medical treatment interfered with your social activities?	1	2	3	4
28. Has your physical condition or medical treatment caused you financial difficulties?	1	2	3	4

For the following questions please circle the number between 1 and 7 that best applies to you

29. How would you rate your overall health during the past week?

1 2 3 4 5 6 7

Very poor Excellent

30. How would you rate your overall quality of life during the past week?

1 2 3 4 5 6 7

Very poor Excellent

SPECIMEN

© Copyright 1995 EORTC Quality of Life Group. All rights reserved. Version 3.0

6. Study-specific questionnaire – preoperative

Name sticker

Pineal Cyst Questionnaire – BEFORE OPERATION

The information that you provide will remain strictly confidential.

Are you currently at work/school? ☐ yes ☐ no

If no, when did you last work? …………………………………………………………………

What is your job? ……………………………………………………………………………………….

Is it full time or part time job? ☐ yes ☐ no

If you are off work, is this because of the symptoms you came to see me with?
☐ yes ☐ no

Weight

- now …… kg
- 1 year ago …… kg (approximately if not known precisely)
- 5 years ago or before symptoms started …… kg (approximately if not known precisely)

Do you think that there has been a relationship between weight and symptoms (presence of the symptoms or their severity)? ☐ yes ☐ no
If yes, please, describe.

Medications

	Name	Started*	Stopped*
HRT			
Contraceptive pill			
Hormonal supplements			
Vitamins			
Painkillers			

*Approximate date when you started and stopped taking this medication.

Have you had glandular fever (= infectious mononucleosis)

☐ yes ☐ no

Headache

Do you suffer from more than one type of headache (that are different in nature)? Please describe.

☐ yes ☐ no

Do you suffer from migraines?

☐ yes ☐ no

Do you have constant headache, i.e. headache that is there all the time and never really goes away? ☐ yes ☐ no

If yes, when did it start? …………..years …………..months ago

Where in the head is your 'main headache'? (tick whichever apply to you)

- ☐ Behind the eye – Left/Right
- ☐ Behind the eyes
- ☐ Between the eyes
- ☐ On the back of the head
- ☐ It is like a tight belt
- ☐ On top of the head
- ☐ In the centre of the head
- ☐ Other (please describe)

Which word best describes your 'main headache'?

- ☐ Sharp
- ☐ Like a tight belt/band
- ☐ Like a knife
- ☐ Pulsating
- ☐ Burning
- ☐ Other (please describe)

When did the **'main headache'** start?

Please give an approximate date

Please give an event, if there is one, that the headaches relate to

Did you 'main headache' start before my migraine headaches? ☐ yes ☐ no ☐ n/a

Does anything trigger your **'main headache'**?

- ☐ Sound/noise
- ☐ Looking up
- ☐ Light
- ☐ Other (please describe)

What makes your **'main headache'** worse?

- ☐ Lying flat
- ☐ Sitting up
- ☐ Working on a computer
- ☐ Any light
- ☐ Fluorescent light
- ☐ Noise
- ☐ Other (list as many causes as you want)

When is your **'main headache'** worst?

- ☐ In the morning
- ☐ In the evening
- ☐ Any other time
- ☐ There is no specific time of day when the headache tends to be worse than at other times

When is your **'main headache'** least bad?

- ☐ In the morning
- ☐ In the evening
- ☐ Any other time
- ☐ There is no specific time of day when the headache tends to be better than at other times

Vision

Please tick all boxes next to the statements that apply to you

- ☐ Blurred vision
- ☐ Double vision
- ☐ When turning my head I experience 'delay in seeing the object in front of me'
- ☐ Tired eyes all the time

What makes your vision-related symptoms worse?

- ☐ Light
  - ☐ Daylight
  - ☐ Fluorescing light
  - ☐ Computer light
  - ☐ Describe
- ☐ Eye movement
  - ☐ Looking to the left
  - ☐ Looking to the right
  - ☐ Looking up
  - ☐ In any direction (is there any direction that makes things worse)

Time of day. My vision related problems are worse

- ☐ Towards the end of the day
- ☐ In the morning
- ☐ At night

Do you have any problems when looking up? ☐ yes ☐ no

If yes – what happens/what symptoms you may get?

Balance problems - dizziness

Which of these statements best describes your symptoms?

- ☐ I have a sensation of movement - as if I was on a rotating chair/the room is spinning
- ☐ My legs feel week
- ☐ I feel like have no control of my legs
- ☐ I feel light-headed

Time profile

- ☐ It is there all the time
- ☐ It starts suddenly
- ☐ It comes and goes. It lasts for
  - ☐ seconds ☐ minutes ☐ hours ☐ days

It is triggered by movement – describe what sort of movement(s)

- ☐ Getting up
- ☐ Suddenly moving my head sideways
- ☐ Bending my head backwards
- ☐ Stress
- ☐ Loud noise
- ☐ Closing eyes

Is anything that makes your balance worse?

- ☐ Getting up
- ☐ Suddenly moving my head sideways
- ☐ Turning my head backwards
- ☐ Stress
- ☐ Loud noise
- ☐ Closing eyes

Hearing

- ☐ Reduced hearing problems on the
  - ☐ left ☐ right ☐ both sides
- ☐ Hearing noises
- ☐ Hearing voices

Memory

Any memory problems? ☐ yes ☐ no

Please describe

Speaking

Any speech problems? ☐ yes ☐ no

Please describe

Concentration

Any problems ☐ yes ☐ no

Please describe

Sleep problems

- ☐ I sleep too much
- ☐ I sleep too little
- ☐ Other sleep problems. Please describe

Other important symptoms

Please list and describe

Have you been seen by an ENT surgeon? ☐ yes ☐ no

What was their diagnosis/explanation?

What treatment did they recommend?

Have you been seen by an ophthalmologist? ☐ yes ☐ no

What was their diagnosis/explanation?

What treatment did they recommend?

Have you been seen by a neurologist? ☐ yes ☐ no

What was their diagnosis/explanation?

What treatment did they recommend?

7. Study-specific questionnaire – 3-month postoperative

Name sticker

Pineal Cyst Questionnaire – AFTER OPERATION
at 3 months

The information that you provide will remain strictly confidential.

As compared to before the operation, overall, I am:

☐ better ☐ worse ☐ no different
☐ much better ☐ much worse

Are you currently at work/school? ☐ yes ☐ no

Your weight …… kg

Medications you currently take

	Name	Started	Stopped
HRT			
Contraceptive pill			
Hormonal supplements			
Vitamins			
Painkillers			

Headache
Please tick the box that best describes this symptom after the operation when compared to how it was before the operation.

☐ better ☐ worse ☐ no different
☐ much better ☐ much worse

☐ I no longer have this symptom ☐ I have never had this symptom

Vision
Please tick the box that best describes this symptom after the operation when compared to how it was before the operation.

☐ better ☐ worse ☐ no different
☐ much better ☐ much worse

☐ I no longer have this symptom ☐ I have never had this symptom

Hearing
Please tick the box that best describes this symptom after the operation when compared to how it was before the operation.

☐ better ☐ worse ☐ no different
☐ much better ☐ much worse

☐ I no longer have this symptom ☐ I have never had this symptom

Dizziness/balance problems
Please tick the box that best describes this symptom after the operation when compared to how it was before the operation.

☐ better ☐ worse ☐ no different
☐ much better ☐ much worse

☐ I no longer have this symptom ☐ I have never had this symptom

Memory
Please tick the box that best describes this symptom after the operation when compared to how it was before the operation.

☐ better ☐ worse ☐ no different
☐ much better ☐ much worse

☐ I no longer have this symptom ☐ I have never had this symptom

Speaking

Please tick the box that best describes this symptom after the operation when compared to how it was before the operation.

☐ better ☐ worse ☐ no different
☐ much better ☐ much worse

☐ I no longer have this symptom ☐ I have never had this symptom

Concentration

Please tick the box that best describes this symptom after the operation when compared to how it was before the operation.

☐ better ☐ worse ☐ no different
☐ much better ☐ much worse

☐ I no longer have this symptom ☐ I have never had this symptom

Sleep problems

Please tick the box that best describes this symptom after the operation when compared to how it was before the operation.

☐ better ☐ worse ☐ no different
☐ much better ☐ much worse

☐ I no longer have this symptom ☐ I have never had this symptom

Other symptoms that I had before the operation

Please tick the box that best describes this symptom after the operation when compared to how it was before the operation. Please name the symptom and use one of the descriptions from below:

Symptom 1:
☐ better ☐ worse ☐ no different
☐ much better ☐ much worse

☐ I no longer have this symptom

Symptom 2:
☐ better ☐ worse ☐ no different
☐ much better ☐ much worse

☐ I no longer have this symptom

Symptom 3:

☐ better	☐ worse	☐ no different
☐ much better	☐ much worse	

☐ I no longer have this symptom

New symptoms that appeared after the operation

Please tick the box that best describes this symptom after the operation when compared to how it was before the operation.

☐ I have the following problems after the operation that I did not have before the operation

Problem/symptom	score: 1-10*

*Please indicate the degree of the symptom, i.e. score it based on how much of a problem it is to you. “1” – it is there, but it is hardly a problem; “10” – it has made my life unbearable

☐ I have no problems that I can relate to the operation

8. Study-specific questionnaire – 12-month postoperative

Name sticker

Pineal Cyst Questionnaire – AFTER OPERATION
at 12 months

The information that you provide will remain strictly confidential.

As compared to before the operation, overall, I am:

☐ better ☐ worse ☐ no different
☐ much better ☐ much worse

Are you currently at work/school? ☐ yes ☐ no

Your weight …… kg

Medications you currently take

	Name	Started	Stopped
HRT			
Contraceptive pill			
Hormonal supplements			
Vitamins			
Painkillers			

Headache

Please tick the box that best describes this symptom after the operation when compared to how it was before the operation.

☐ better ☐ worse ☐ no different
☐ much better ☐ much worse

☐ I no longer have this symptom ☐ I have never had this symptom

Vision

Please tick the box that best describes this symptom after the operation when compared to how it was before the operation.

☐ better ☐ worse ☐ no different
☐ much better ☐ much worse

☐ I no longer have this symptom ☐ I have never had this symptom

Hearing

Please tick the box that best describes this symptom after the operation when compared to how it was before the operation.

☐ better ☐ worse ☐ no different
☐ much better ☐ much worse

☐ I no longer have this symptom ☐ I have never had this symptom

Dizziness/balance problems

Please tick the box that best describes this symptom after the operation when compared to how it was before the operation.

☐ better ☐ worse ☐ no different
☐ much better ☐ much worse

☐ I no longer have this symptom ☐ I have never had this symptom

Memory

Please tick the box that best describes this symptom after the operation when compared to how it was before the operation.

☐ better ☐ worse ☐ no different
☐ much better ☐ much worse

☐ I no longer have this symptom ☐ I have never had this symptom

Speaking

Please tick the box that best describes this symptom after the operation when compared to how it was before the operation.

☐ better ☐ worse ☐ no different
☐ much better ☐ much worse

☐ I no longer have this symptom ☐ I have never had this symptom

Concentration

Please tick the box that best describes this symptom after the operation when compared to how it was before the operation.

☐ better ☐ worse ☐ no different
☐ much better ☐ much worse

☐ I no longer have this symptom ☐ I have never had this symptom

Sleep problems

Please tick the box that best describes this symptom after the operation when compared to how it was before the operation.

☐ better ☐ worse ☐ no different
☐ much better ☐ much worse

☐ I no longer have this symptom ☐ I have never had this symptom

Other symptoms that I had before the operation

Please tick the box that best describes this symptom after the operation when compared to how it was before the operation. Please name the symptom and use one of the descriptions from below:

Symptom 1:
☐ better ☐ worse ☐ no different
☐ much better ☐ much worse

☐ I no longer have this symptom

Symptom 2:
☐ better ☐ worse ☐ no different

☐ much better ☐ much worse

☐ I no longer have this symptom

Symptom 3:
☐ better ☐ worse ☐ no different
☐ much better ☐ much worse

☐ I no longer have this symptom

New symptoms that appeared after the operation
Please tick the box that best describes this symptom after the operation when compared to how it was before the operation.

☐ I have the following problems after the operation that I did not have before the operation

Problem/symptom	score: 1-10*

*Please indicate the degree of the symptom, i.e. score it based on how much of a problem it is to you. "1" – it is there, but it is hardly a problem; "10" – it has made my life unbearable

☐ I have no problems that I can relate to the operation
