# Supplementary material for: Surgery for pineal cysts with symptoms in the absence of hydrocephalus: a prospective cohort study
Source: eClinicalMedicine. 2025 Sep 19;89:103514. doi: 10.1016/j.eclinm.2025.103514 (PMC12495433; doi:10.1016/j.eclinm.2025.103514)
Supplement: Supplementary Figures [file mmc2.pptx]

## Slide 1
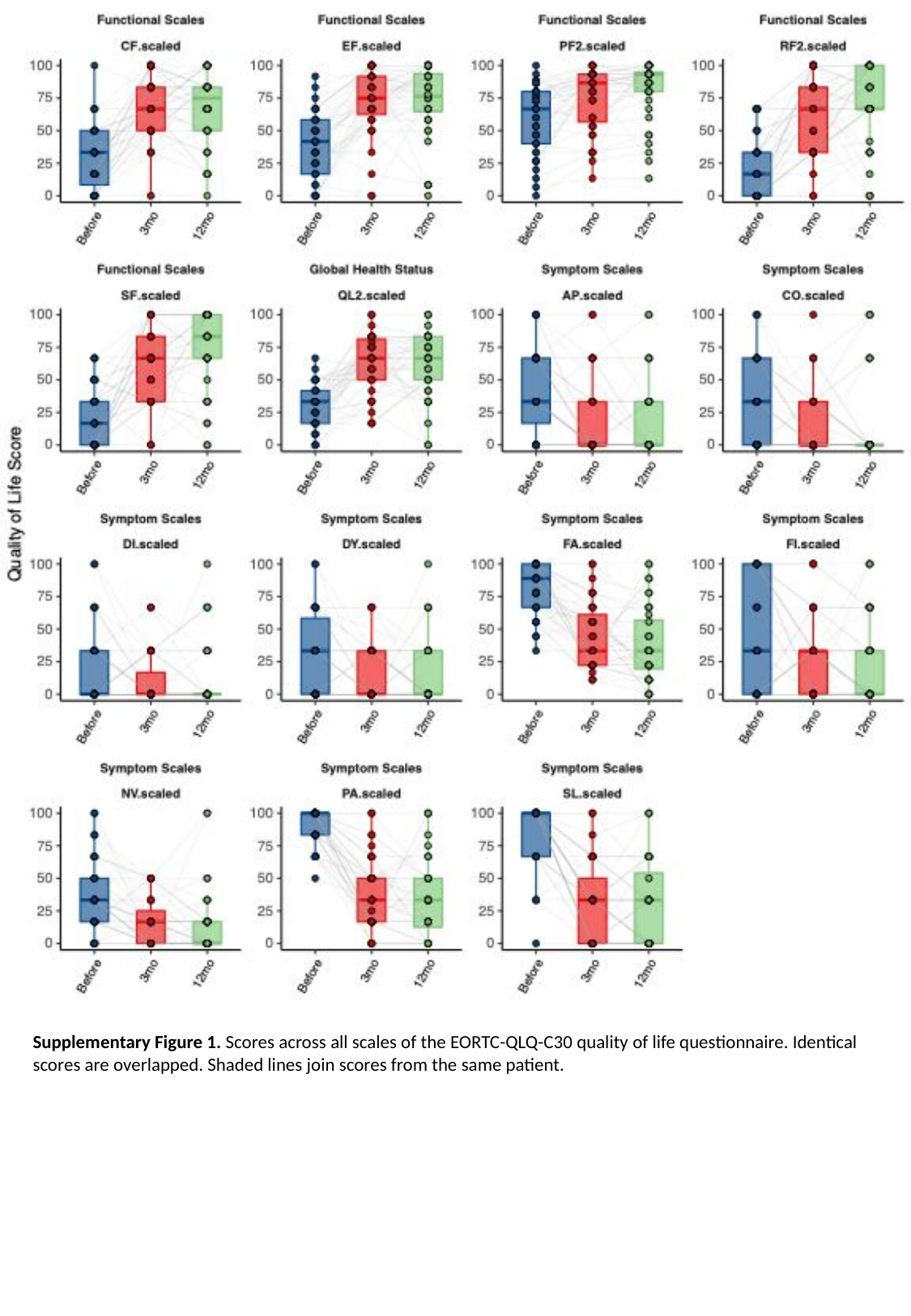

Supplementary Figure 1. Scores across all scales of the EORTC-QLQ-C30 quality of life questionnaire. Identical scores are overlapped. Shaded lines join scores from the same patient.

## Slide 2
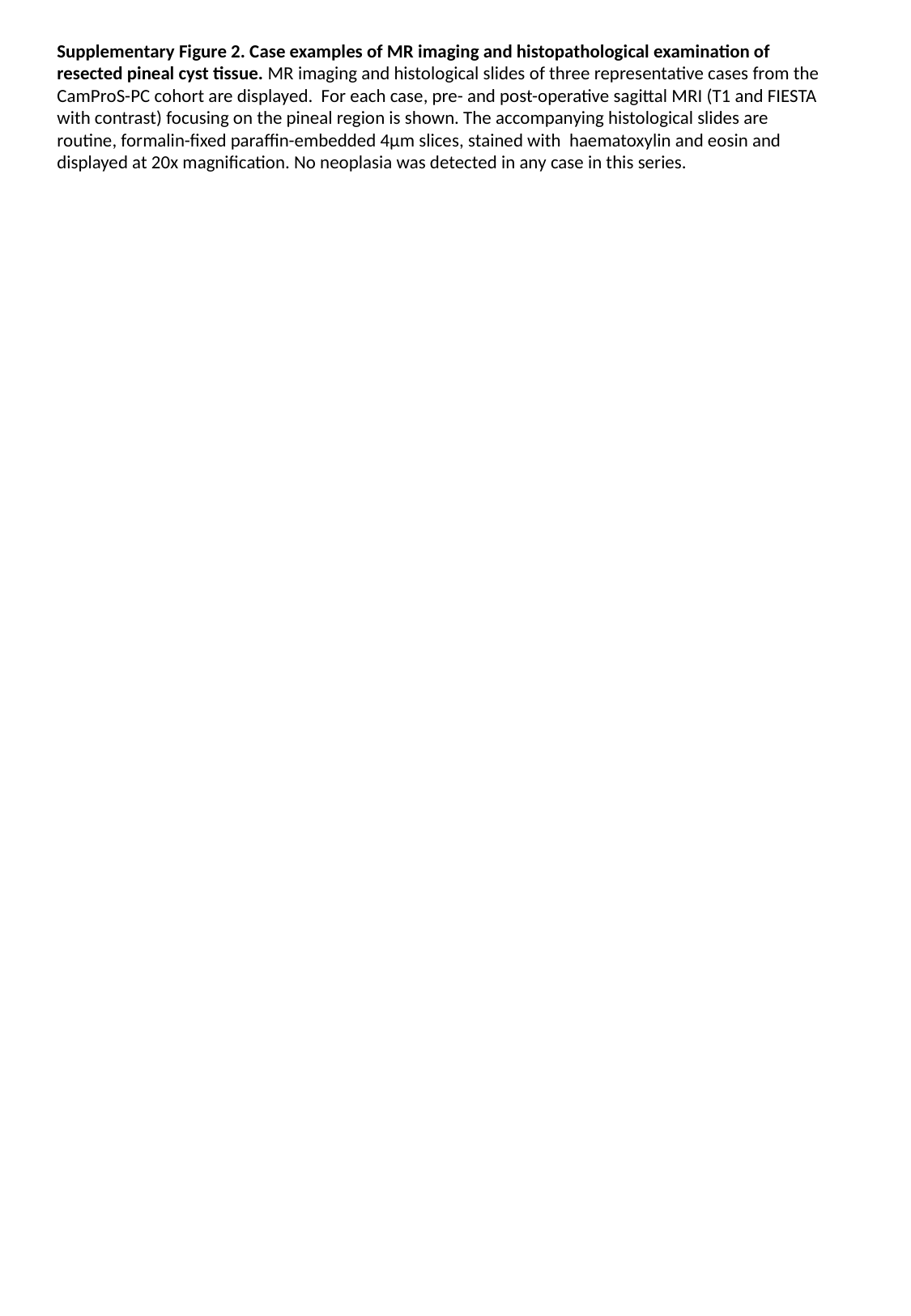

Supplementary Figure 2. Case examples of MR imaging and histopathological examination of resected pineal cyst tissue. MR imaging and histological slides of three representative cases from the CamProS-PC cohort are displayed. For each case, pre- and post-operative sagittal MRI (T1 and FIESTA with contrast) focusing on the pineal region is shown. The accompanying histological slides are routine, formalin-fixed paraffin-embedded 4µm slices, stained with haematoxylin and eosin and displayed at 20x magnification. No neoplasia was detected in any case in this series.

## Slide 3
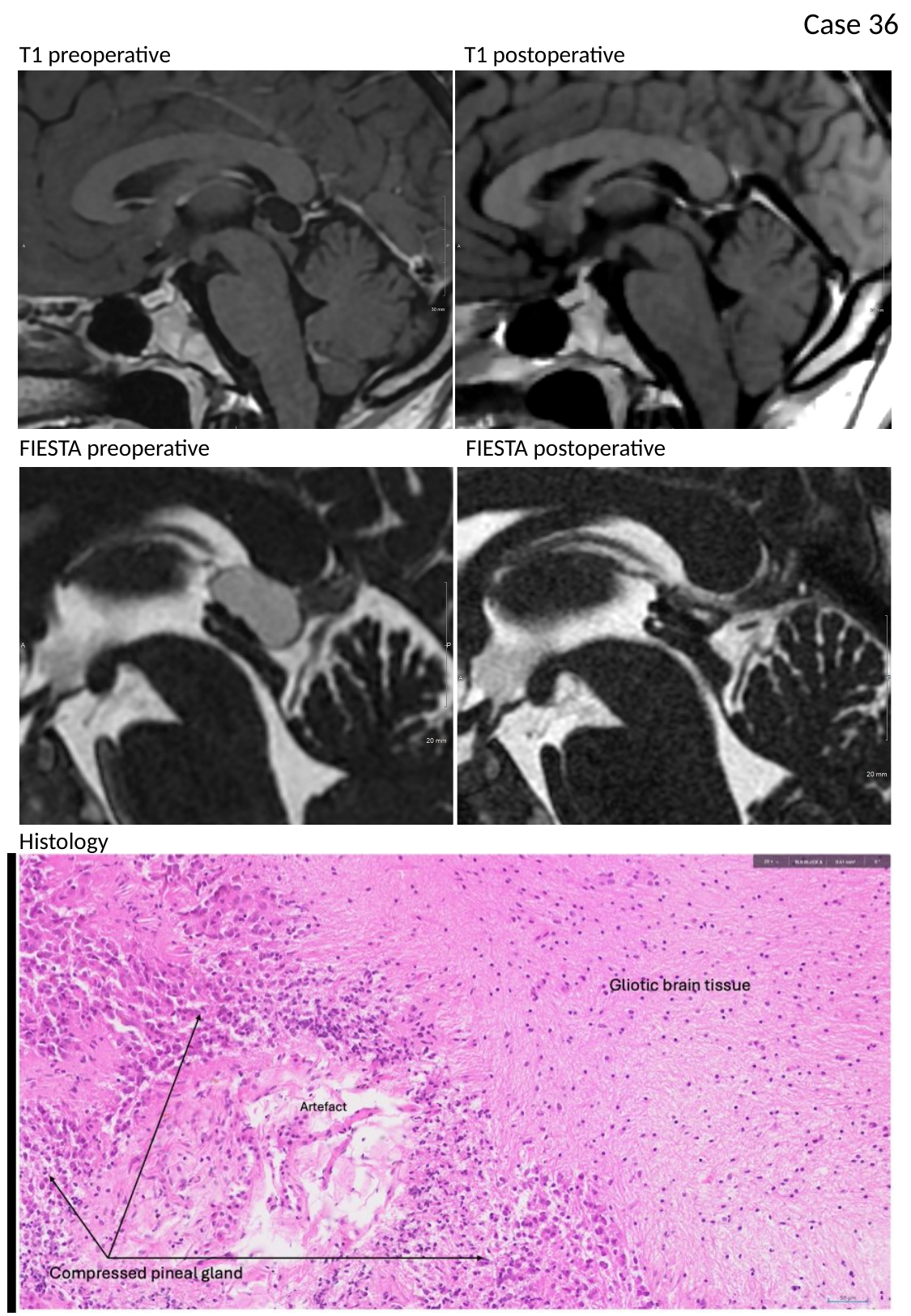

Case 36
T1 postoperative
T1 preoperative
Case 36
FIESTA postoperative
FIESTA preoperative
Histology

## Slide 4
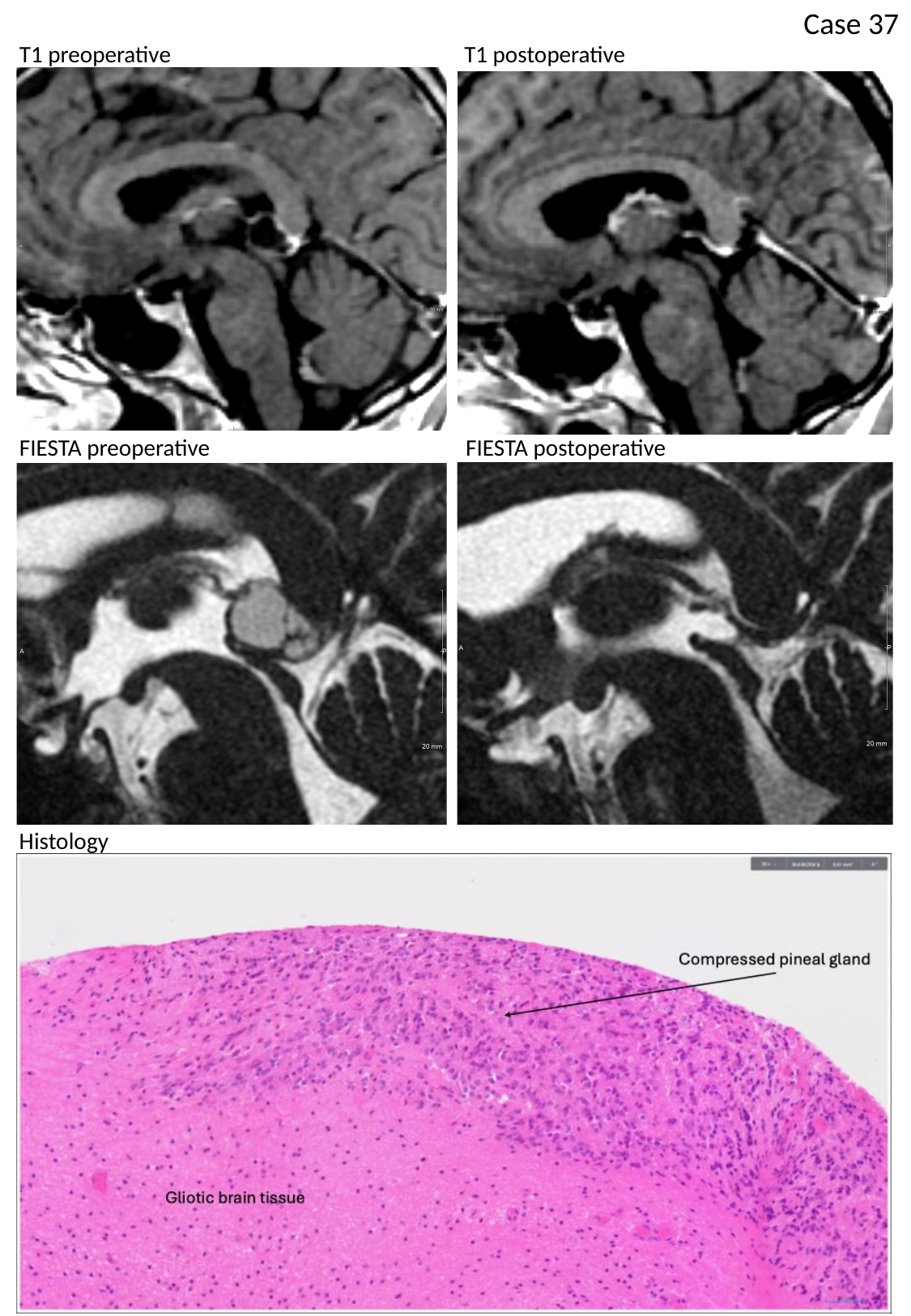

Case 37
T1 postoperative
T1 preoperative
Case 36
FIESTA postoperative
FIESTA preoperative
Histology

## Slide 5
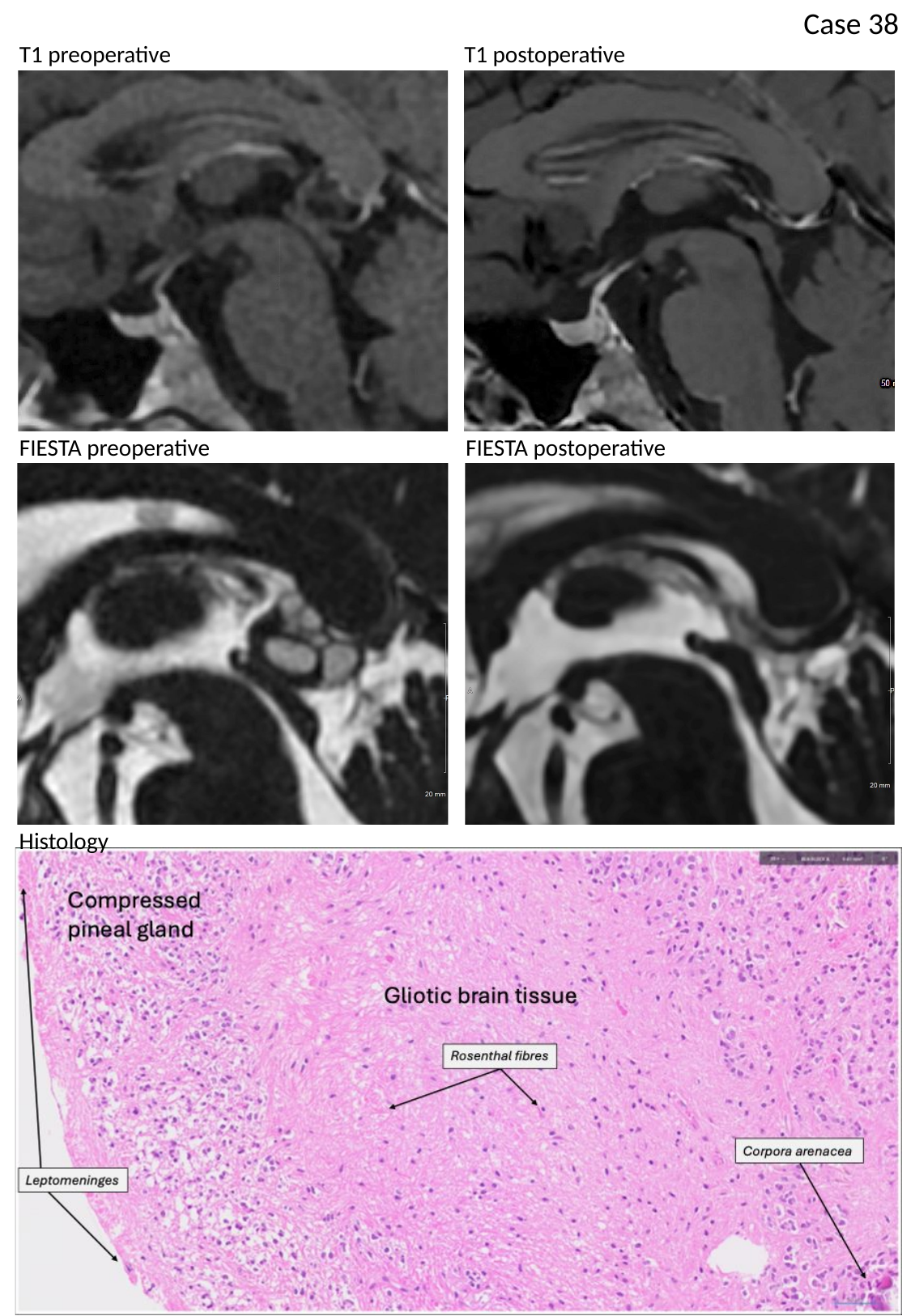

Case 38
T1 postoperative
T1 preoperative
Case 36
FIESTA postoperative
FIESTA preoperative
Histology
